# Fluorescent copolymer aggregate sensor for lithium chloride†

**DOI:** 10.1039/d2sc05342j

**Published:** 2023-03-22

**Authors:** Hu Wang, Leighton O. Jones, Tian Zhao, Inhong Hwang, Vincent M. Lynch, Niveen M. Khashab, George C. Schatz, Zachariah A. Page, Jonathan L. Sessler

**Affiliations:** a Department of Chemistry, The University of Texas at Austin 105 East 24th Street, Stop A5300 Austin Texas 78712 USA sessler@cm.utexas.edu zpage@cm.utexas.edu; b Department of Chemistry, Northwestern University Evanston Illinois 60208-3113 USA g-schatz@northwestern.edu; c Smart Hybrid Materials (SHMs) Laboratory, Advanced Membranes and Porous Materials Center, King Abdullah University of Science and Technology Thuwal Saudi Arabia niveen.khashab@kaust.edu.sa

## Abstract

We report a copolymeric fluorescent sensor that is selective for lithium chloride. The two constituent polymers comprise pendent triphenylethylene (TPE) moieties for aggregate induced emission (AIE) along with either strapped-calix[4]pyrrole or secondary ammonium groups that drive aggregation *via* self-assembly upon polymer mixing. Addition of LiCl in acetonitrile disrupts the strapped-calix[4]pyrrole/secondary ammonium chloride salt host–guest crosslinks leading to disaggregation of the polymer chains and a decrease in TPE emission. The lack of AIE perturbation upon addition of NaCl, KCl, MgCl_2_ or CaCl_2_ provides for high selectivity for LiCl relative to potential interferants. This supramolecular dual polymer approach could serve as a complement to more traditional sensor systems.

## Abstract 2

We report a copolymeric lithium chloride selective fluorescent sensor. No fluorescence change is seen upon the addition of NaCl, KCl, MgCl_2_, or CaCl_2_, while a fluorescence decrease is seen upon the addition of LiCl.

## Graphical abstract

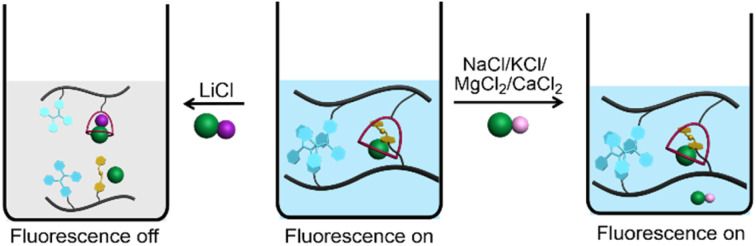

## Introduction

Lithium is critical to the lithium-ion battery industry and thus plays a foundational role in the evolving development of electronic products,^1–9^ such as computers, digital cameras, mobile phones, and mobile power tools. A consequence is an increase in industrial and consumer waste, which can lead to the leaching of lithium into waterways and wells causing a commensurate increase in the lithium content of drinking water. While lithium provides a therapeutic effect against certain mental health disorders,^10–14^ excessive lithium can be harmful;^15^ it can cause irritation to the skin, eyes, and respiratory tract, and affect adversely the central nervous system and inducing kidney damage. As such, treatment protocols and environmental monitoring would benefit from facile lithium sensing.

Current lithium detection methods include flame tests,^16^ spectroscopic methods,^17^ and electrochemical analyses.^18,19^ Fluorescence sensors offer the potential for high sensitivity, good selectivity, and ease-of-use; they thus occupy a time-honored role in analytical chemistry and biology.^20–24^ Unfortunately, at present fluorescent-based strategies for lithium detection are still in their infancy. In 2015, Novakova and co-workers reported a series of fluorescence sensors for alkali metal cations (Li^+^, Na^+^, K^+^) and alkaline earth metal cations (Mg^2+^, Ca^2+^, Ba^2+^) based on crown ethers as the recognition moieties and an aza-analogue of phthalocyanine as the fluorophore.^25^ In separate work, Ji and co-workers developed two fluorescent probes, which proved capable of measuring two different metal ions under basic and acidic conditions, respectively.^26^ Suzuki and co-workers reported several fluorescent Li^+^ chemosensors (Li^+^ detection limit: 0.6 mM or 4.1 ppm) that allowed quantitative measurements of lithium in clinical samples,^27^ although possible interference from Na^+^ was noted at the lower therapeutic Li^+^ levels. Here we report a copolymer-based lithium sensor that permits LiCl detection in acetonitrile *via* fluorescence modulation with little interference from other test salts, including NaCl, KCl, MgCl_2_, and CaCl_2_. This system differs from more classic fluorescent sensors in that it does not involve a receptor subunit tethered to a fluorophore. Rather, it relies on two separate polymer chains containing triphenylethylene (TPE) subunits whose aggregation-induced emission (AIE) intensity is controlled through LiCl-mediated inter-chain host–guest interactions.

The first report of AIE was by Tang, *et al.* in 2001.^28–31^ One of the most widely studied AIEgens (compounds producing an AIE response) is triphenylethylene (TPE), which is highly luminescent in the aggregated state as the result of restriction of intramolecular rotation (RIR). To date, TPE has been exploited for cell imaging,^32,33^ fluorescent sensor development,^34,35^ and mechanafluorochromic materials chemistry.^36^ However, to our knowledge TPE and AIE effects have yet to be applied to lithium sensing.

## Results and discussion

**Scheme 1:**
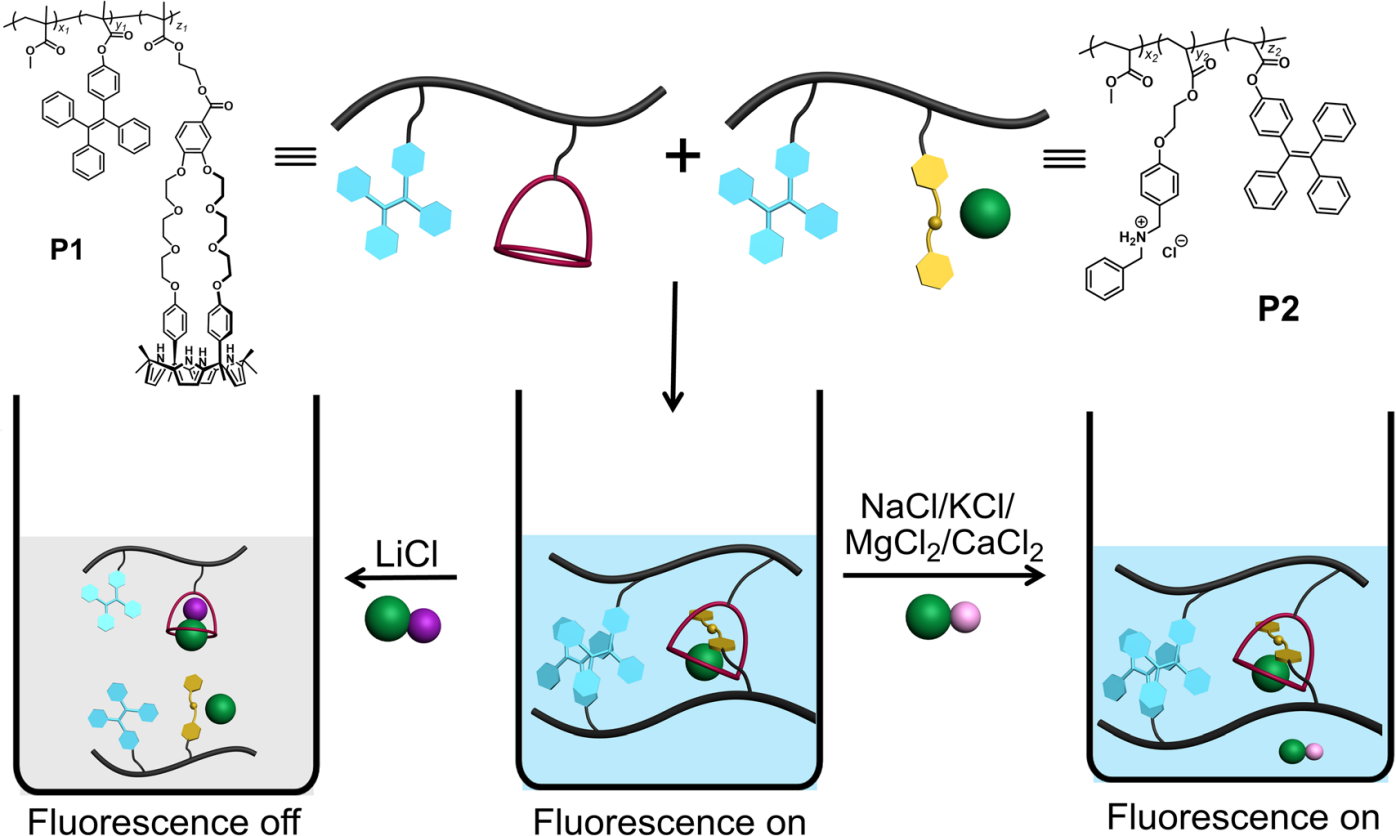
Chemical structures of P1 and P2. Also provided is an illustration of P1 and P2 self-assembly in acetonitrile, along with the selective effect of LiCl to induce disaggregation and a loss of fluorescence.

**Fig. 1:**
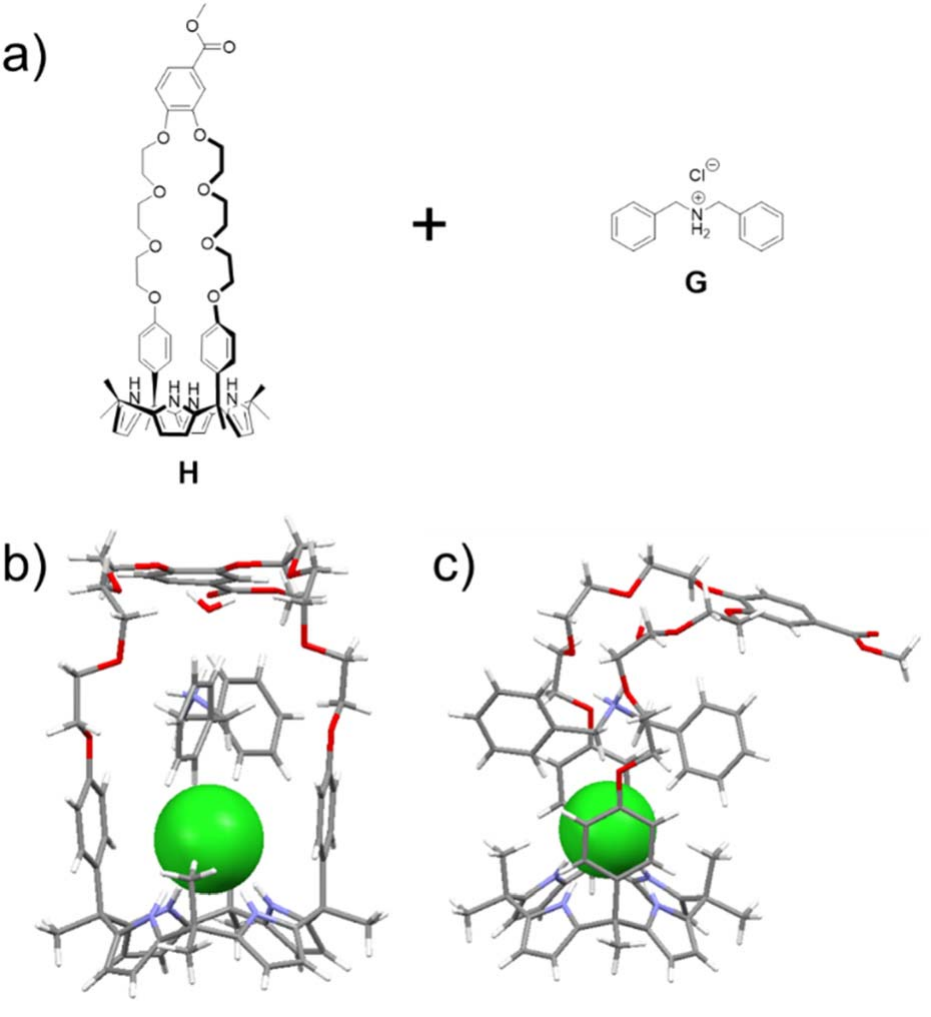
(a) Chemical structures of H and G; (b) front and (c) side views of a single crystal X-ray diffraction structure of the H⊃G complex. The chloride anion is shown in green. A bound water and solvents present in the crystal lattice have been omitted for clarity.

Scheme 1 shows our approach to creating a fluorescent LiCl sensor. It is predicated on the use of supramolecular polymer aggregates. The polymer aggregates were constructed from P1, a polymer containing a crown ether strapped-calix[4]pyrrole H and TPE subunits as pendent groups, and polymer P2 bearing a secondary ammonium chloride salt G and TPE substituents. Mixing P1 and P2 leads to cross-linking between the polymer chains as the result of host–guest interactions between H and G (see Fig. 1 for structures). In contrast to the individual polymer chains in solution, the mixed system proved fluorescent, presumably as the result of TPE-based AIE. Solution phase studies revealed that in acetonitrile the interaction between H and LiCl is greater than that between H and G. LiCl thus promotes disaggregation of the two polymer chains and concomitant fluorescence quenching. On the other hand, NaCl, KCl, MgCl_2_ or CaCl_2_ were not found to bind H well or act as interferants when the mixture of P1 and P2 in acetonitrile is used as a LiCl sensor.

A single crystal X-ray diffraction analysis provided support for the proposed host–guest interactions between H and G (Fig. 1). Single crystals of H⊃G complex were obtained by allowing diisopropyl ether to diffuse slowly into a mixed solution of H and G in acetonitrile. The resulting structure revealed that complex H⊃G adopts a [2]pseudorotaxane threaded structure in the solid state. The secondary ammonium cation is stabilized within the crown ether cavity *via* presumed N^+^–H⋯O hydrogen bonding interactions while the Cl^−^ anion in H⊃G is stabilized by four hydrogen bond interactions with the pyrrole NH protons as typical for a calix[4]pyrrole anion complex.^37,38^

Support for the notion that LiCl would out-compete G for H comes from calculations. In our previous study^37^ we considered four limiting conformations for the interaction of H with representative metal chloride salts. In this study, we selected two distinct conformations, specifically the *exo* and *endo* conformers, which were previously labelled as isomers 2 and 6;^37^ these two conformers differ by the position of the arm with respect to the receptor body, either pointing up from the crown moiety or down and parallel to it (*cf.* Fig. S32† for structural illustrations).

**Fig. 2:**
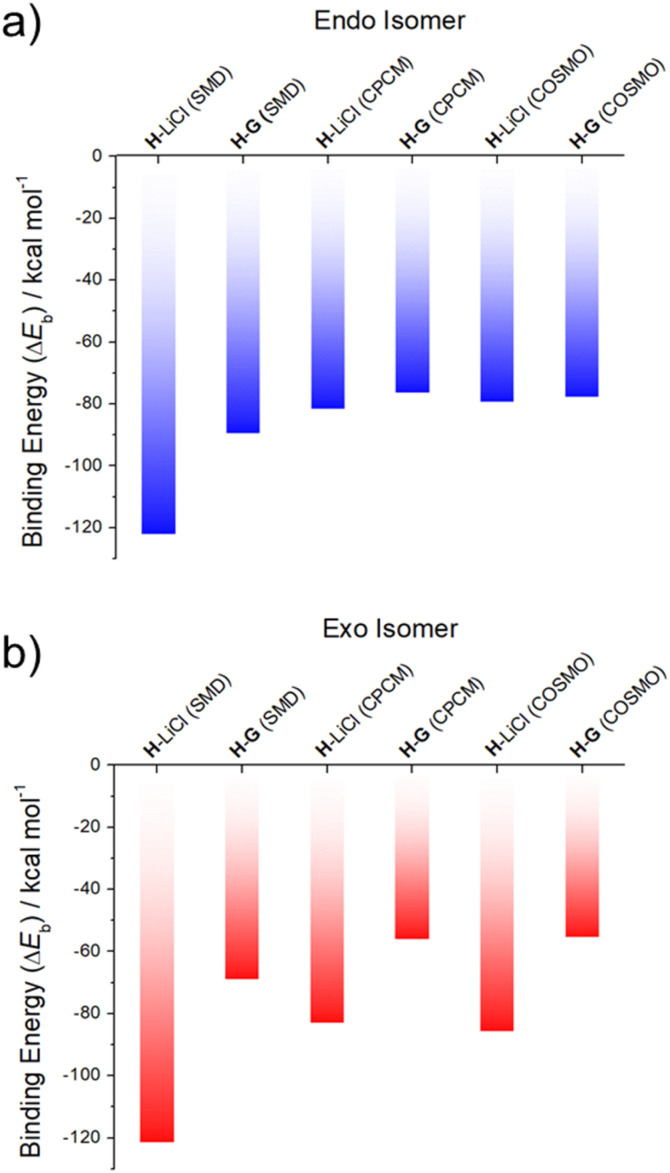
DFT-calculated binding energies of LiCl (from ref. 37) and G to two different conformational isomers of the receptor H: (a) *endo* and (b) *exo*, in SMD, CPCM and COSMO acetonitrile solvent continuums.

As can be seen from an inspection of Fig. 2, G is calculated to bind to H less well than does LiCl. As such, we deemed it likely that G would be replaced by LiCl under conditions of thermodynamic control. The underlying calculations were carried out using three common solvent continuum models, namely SMD, CPCM and COSMO, all of which reproduce the favorability of LiCl over G. The preference for LiCl is more pronounced in the case of the *exo* isomer, a finding ascribed to intramolecular interactions that dampen the difference in the binding energies of the salts (*i.e.*, LiCl *vs.*G) in the case of the *endo* isomer.

**Fig. 3:**
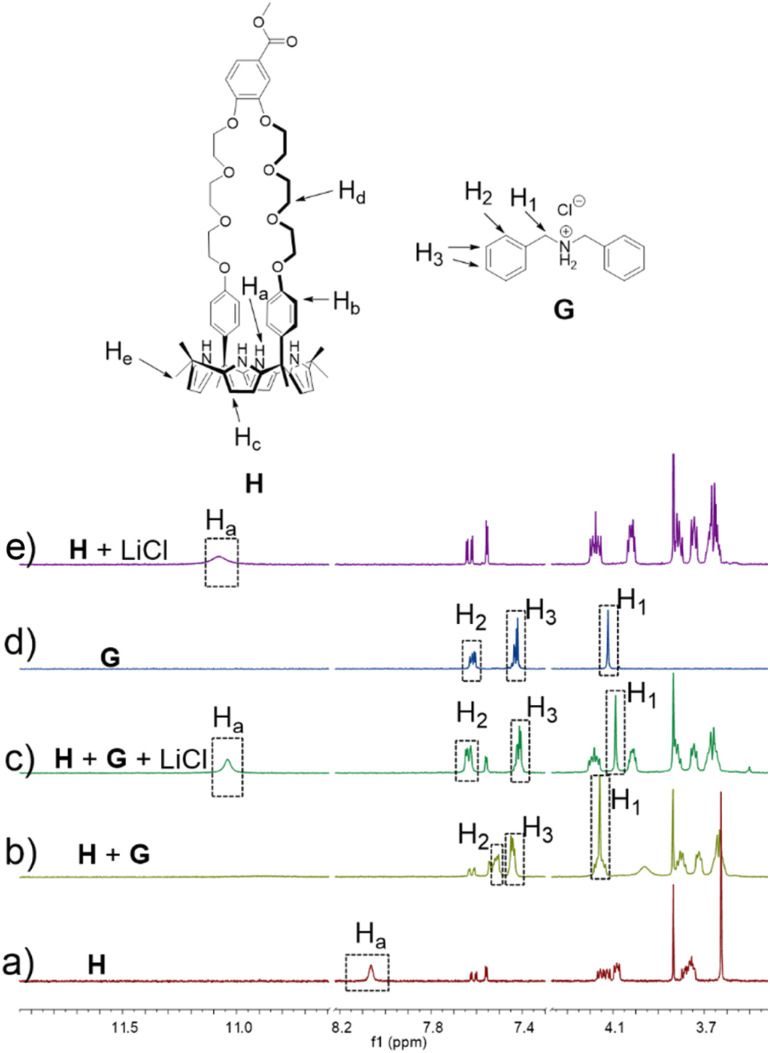
Partial ^1^H NMR spectra showing spectral changes for different combinations of H, G, and LiCl (400 MHz, CD_3_CN, 295 K): (a) H (5.00 mM); (b) H (5.00 mM) and G (5.00 mM); (c) after addition of 5.00 mM LiCl to (b and d) G (5.00 mM); (e) H (5.00 mM) and LiCl (5.00 mM).

To test whether H would interact with G in solution, both species (5.00 mM in each) were mixed in acetonitrile-*d*_3_. Upon mixing, spectral changes consistent with the formation of a H⊃G complex analogous to that seen in the solid state were observed (Fig. 3). A quantitative ^1^H NMR spectral titration led to a calculated *K*_a_ of (1.4 ± 0.13) × 10^4^ M^−1^ for the interaction between H and G in acetonitrile-*d*_3_ (Fig. S17†). A 2D NOESY spectrum (Fig. S15†) of a mixture of 15.0 mM H and 15.0 mM G in acetonitrile-*d*_3_ revealed correlations between protons H_d_ of H and protons H_1_ on G, as would be expected for a structure wherein G is threaded into the cavity of H.

The formation of H⊃G under model conditions led us to suggest that the underlying interactions would stabilize an association between P1 and P2 and that the resulting aggregated state would support an AIE-based fluorescence from the constituent TPE subunits. The design expectation was that the crosslinking interactions between P1 and P2 would be broken up selectively *via* contact with LiCl, leading to disaggregation of TPE subunits to a non-fluorescent state. In a previous study, we found that H binds LiCl with high affinity (*K*_a_ = 2 × 10^5^ M^−1^ in acetonitrile) and that potentially competing salts, such as NaCl, KCl, MgCl_2_, and CaCl_2_, were not complexed effectively.^37^ Unknown was whether LiCl would outcompete G. To test this, LiCl (5.00 mM) was added to a 1 : 1 mixture of H (5.00 mM) and G (5.00 mM) in acetonitrile-*d*_3_. ^1^H NMR spectral features analogous to those of a bona fide sample of H⊃LiCl were seen (Fig. 3). We thus infer that the interaction between LiCl and H in acetonitrile exceeds that between G and H. A similar NMR spectral analysis revealed no change in the chemical shifts of a 1 : 1 mixture of H (5.00 mM) and G (5.00 mM) upon adding NaCl (0.29 g L^−1^), KCl (0.37 g L^−1^), MgCl_2_ (0.48 g L^−1^) or CaCl_2_ (0.55 g L^−1^) (Fig. S18–S21†). We thus conclude that NaCl, KCl, MgCl_2_, and CaCl_2_ would not outcompete the G subunits of P2 for the H receptors present in P1. Accordingly, the mixture of P1 and P2 was expected to be a selective “turn off” sensor for LiCl in acetonitrile.

Polymers P1 (*M*_n_ = 30.5 kDa and PDI = 1.56) and P2 (*M*_n_ = 27.2 kDa and PDI = 1.61) were prepared by free radical copolymerization (Fig. S22 and S23†). The size and size distribution of different concentrations of P1, P2 and P1 + P2 in acetonitrile were measured by dynamic light scattering (DLS) (Fig. S24†). The calculated particle sizes for P1 (0.032 mM) and P2 (0.020 mM) were both ∼9 nm, in accord with what is expected for traditional polymer agglomeration. In contrast, the particle size of a mixture of P1 (0.032 mM) + P2 (0.020 mM) is about 44 nm. The formation of these relatively large particles is ascribed to cross-linking arising from interactions between H and G.

Consistent with the above thinking, we found that as the total concentration of the mixture of P1 and P2 in acetonitrile solution was increased, the size of the aggregates likewise increased. The particle size of the higher concentration mixture of P1 (0.16 mM) + P2 (0.10 mM) was found to be about 140 nm *vs. ca.* 10 nm for P1 (0.16 mM) and P2 (0.10 mM) alone (Fig. S25a†). TEM imaging and consideration of the observed microscopic morphologies leads us to conclude that P1 (0.16 mM), P2 (0.10 mM), and a mixture of P1 (0.16 mM) and P2 (0.10 mM), all exist in the form of nanoparticles in acetonitrile (Fig. S25b†). Considering the control studies with H and G (*vide supra*), we postulated that adding LiCl to a mixed solution of P1 (0.16 mM) and P2 (0.10 mM) would destroy the host–guest cross-links, converting large fluorescent aggregates to non-emissive smaller-sized particles (Fig. S25a†).

**Fig. 4:**
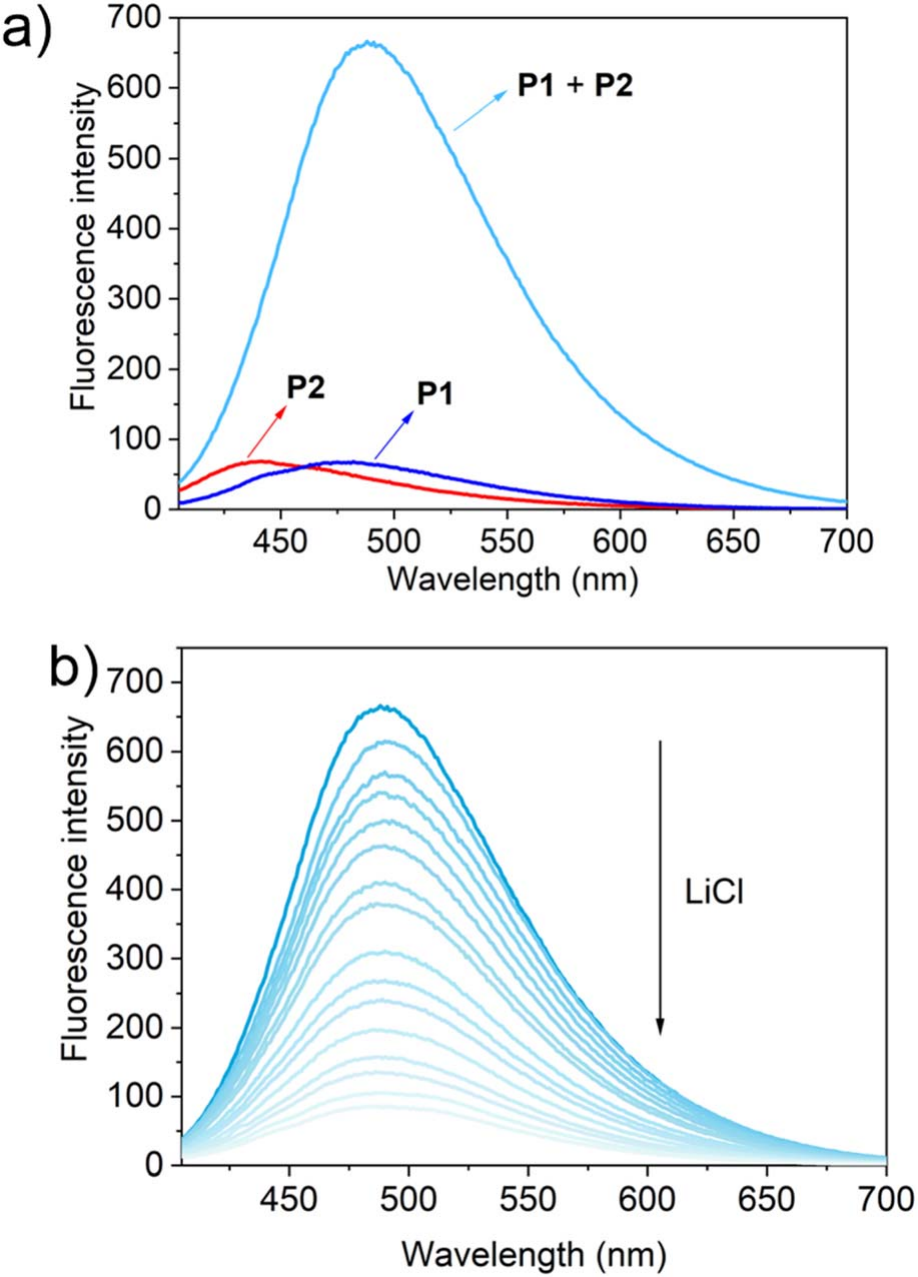
(a) Fluorescence spectra of P1 (1.60 μM), P2 (1.00 μM) and P1 (1.60 μM) + P2 (1.00 μM); (b) the changes in fluorescence intensity of P1 (1.60 μM) + P2 (1.00 μM) upon the titration of LiCl (0.00–150 μM) in acetonitrile (*λ*_ex_ = 380 nm).

**Fig. 5:**
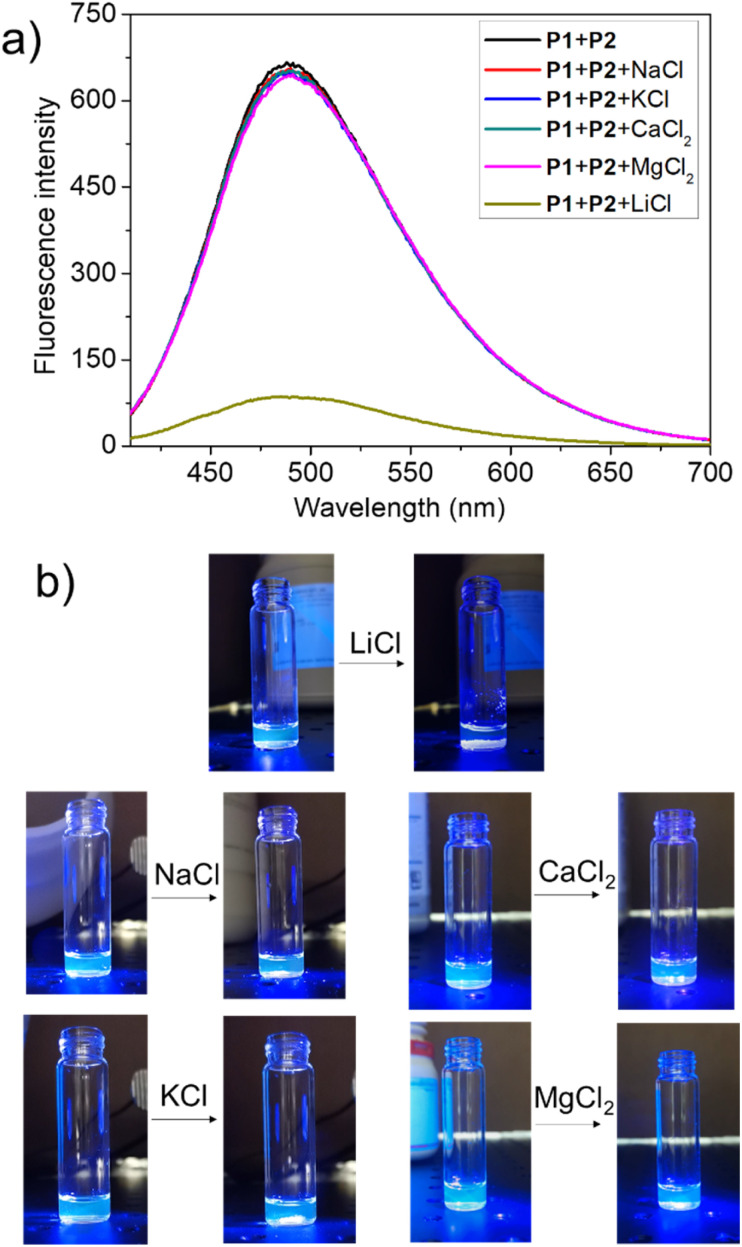
(a) Fluorescence spectra of P1 (1.60 μM) + P2 (1.00 μM) in acetonitrile and after adding excess solid LiCl, NaCl, KCl, MgCl_2_, and CaCl_2_; (b) photos highlighting the change in fluorescence (if any) for a mixture of P1 (1.60 μM) + P2 (1.00 μM) seen upon the addition of excess solid LiCl, NaCl, KCl, MgCl_2_, and CaCl_2_.

To test this hypothesis, the change in the fluorescence intensity of a mixture of P1 (1.60 μM) + P2 (1.00 μM) in acetonitrile was monitored upon titration with LiCl (0.00–150 μM) (*λ*_ex_ = 380 nm). The fluorescence quantum yields of P1 and P2 are 1.3% and 1.5% respectively. The addition of LiCl led to a decrease in the fluorescence intensity of the P1 + P2 mixture (Fig. 4b; Video S1†), a finding attributed to the destruction of host–guest crosslinking by the added LiCl. Based on the extent of quenching observed when P1 (1.60 μM) was titrated with P2 (1.00 μM) in acetonitrile (Fig. S26†), we conclude that the limit of detection is 4.5 × 10^−7^ M (3.1 ppb). When excess solid NaCl, KCl, MgCl_2_ or CaCl_2_ were added, the fluorescence intensity of the solution did not decrease significantly (Fig. 5; Videos S2–S5†). The change in fluorescence could also be followed visually. After adding excess solid LiCl and shaking briefly, the P1 + P2 solution changed quickly from cyan to colourless under UV irradiation (*λ*_ex_ = 365 nm). When excess solid NaCl, KCl, MgCl_2_ or CaCl_2_ were added, no appreciable change in colour was observed. Nor, did the presence of these salts interfere with the response produced by LiCl.

## Conclusions

In summary, a fluorescent sensor selective for LiCl in acetonitrile was prepared. This system is based on the finding that the complex between H and LiCl is stronger than that between H and G. Therefore, LiCl can displace G from the H cavity. Introducing H and G into two separate polymer chains provided two AIEgen polymers: P1 with H and TPE as pendent groups and P2 with G and TPE as pendent groups. P1 and P2 formed large aggregates through host–guest interactions in acetonitrile. These large aggregates proved highly fluorescent. This fluorescence is quenched by LiCl, but not by the potential interferants NaCl, KCl, MgCl_2_ and CaCl_2_. Therefore, the combination of P1 and P2 allows for the specific recognition and sensing of LiCl. We suggest that the supramolecular dual polymer approach detailed here could serve as a complement to more traditional sensor systems. Efforts to generalize the present finding are thus underway.

## Data availability

All data associated with this report may be found in the ESI.†

## Author contributions

Conceptualization and supervision: JLS, ZAP, GCS, and NMK; synthesis, characterization, NMR, spectroscopy, TEM, GPC and DLS studies: HW, TZ and IH; single crystal growing and data analysis: HW and VML; DFT calculations: LOJ and GCS; writing – original: HW; writing – review and editing: JLS, ZAP, GCS, and NMK. All authors proofread, commented on, and approved the final version of this manuscript.

## Conflicts of interest

The authors declare no conflict of interest.

## Supplementary Material

SC-014-D2SC05342J-s001

SC-014-D2SC05342J-s002

SC-014-D2SC05342J-s003

SC-014-D2SC05342J-s004
