# Supplementary material for: Fluorescent copolymer aggregate sensor for lithium chloride
Source: Chem Sci. 2023 Mar 22;14(15):4120–5. doi: 10.1039/d2sc05342j (PMC10094405; doi:10.1039/d2sc05342j)
Supplement: SC-014-D2SC05342J-s001 [file SC-014-D2SC05342J-s001.pdf]

Electronic Supplementary Material (ESI) for Chemical Science.
This journal is © The Royal Society of Chemistry 2023

Supporting Information for:

Fluorescent Copolymer Aggregate Sensor for Lithium Chloride

Hu Wang,[a] Leighton O. Jones,[b] Tian Zhao,[a] Inhong Hwang,[a] Vincent M. Lynch,[a] Niveen M. Khashab,*[c] George C. Schatz,*[b] Zachariah A. Page*[a] and Jonathan L. Sessler*[a]

a Department of Chemistry, 105 East 24th Street, Stop A5300, The University of Texas at Austin, Austin, Texas 78712, United States. Email: sessler@cm.utexas.edu; zpage@cm.utexas.edu

b Department of Chemistry, Northwestern University, Evanston, Illinois 60208, United States. Email: g-schatz@northwestern.edu

c Smart Hybrid Materials (SHMs) Laboratory Advanced Membranes and Porous Materials Center King Abdullah University of Science and Technology Thuwal, 23955, Saudi Arabia. E-mail: niveen.khashab@kaust.edu.sa

Supporting Information

1. *Materials and methods* S2
2. *Synthesis of guest monomer **5*** S3
3. *Partial NOESY NMR spectrum (400 MHz, 298 K) of a CD_3CN solution 15.0 mM in **H** and 15.0 mM in **G**.* S16
4. *Determination of the association constant for **H⊃G*** S17
5. *NMR spectral analysis the 1:1 mixture of **H** and **G** recorded in the absence and presence of added NaCl, KCl, $MgCl_2$ and $CaCl_2$* S20
6. *Synthesis of polymers **P1** and **P2*** S23
7. *DLS results and TEM images* S27
8. *Linear correlation between the fluorescence emission intensity at 485 nm of **P1** + **P2** and LiCl concentration* S28
9. *Fluorescence spectra of **P1** (1.60 µM) + **P2** (1.00 µM) in acetonitrile with time after adding excess solid LiCl, NaCl, KCl, $MgCl_2$, and $CaCl_2$* S29
10. *Fluorescence spectra of **P1** (1.60 µM) + **P2** (1.00 µM) in the competitive binding experiment* S30
11. *The instrument parameter conditions of the fluorescence test* S31
12. *X-ray experimental* S32
13. *Computational methods* S37
14. *Computational data* S38
15. *Supplementary references* S41

1. Materials and methods

All reagents and starting materials were obtained from commercial suppliers and used without further purification unless otherwise noted. Compounds **1**[S1] and monomer **7**[S2] were prepared according to published procedures. One-dimensional nuclear magnetic resonance (NMR) spectra were recorded on Agilent MR 400 and Varian Inova 500 instruments. ESI mass spectra were obtained on an Agilent Technologies 6530 Accurate-Mass Q-TOF LC/MS or a Thermo Scientific TSQ Quantum GC/MS. An attenuated total reflection cell equipped with a Ge crystal was employed. Fluorescence measurements were performed on a Perkin-Elmer Luminescence Spectrophotometer LS 50B or a Gilden Photonics Ltd. fluorimeter. Transmission electron microscopy (TEM) studies were carried out on a HITACHI HT-7700 instrument. Gel permeation chromatography (GPC) was performed on a Tosoh EcoSEC HLC-8320 GPC System equipped with a series of TSKgel SuperHM-H, -M, and -N columns. The instrument is equipped with a refractive index (RI) and ultraviolet (UV) absorbance detector (UV-8320). The instrument was run with HPLC-grade tetrahydrofuran as the eluent with toluene as an internal standard, at a flowrate of 0.35 mL/min, temperature of 40 °C, and injection volume of 50-100 μL. Prior to injection, all samples were dissolved fully in the mobile phase at a concentration of ~1-5 mg/mL and passed through a 0.45 μm syringe filter. Molecular weight and molecular weight distributions for the samples were estimated using a calibration curve generated from a set of poly(methyl methacrylate) standards (PstQuick® Kit-H) purchased from Tosoh Bioscience.

2. *Synthesis of guest monomer* ***5***

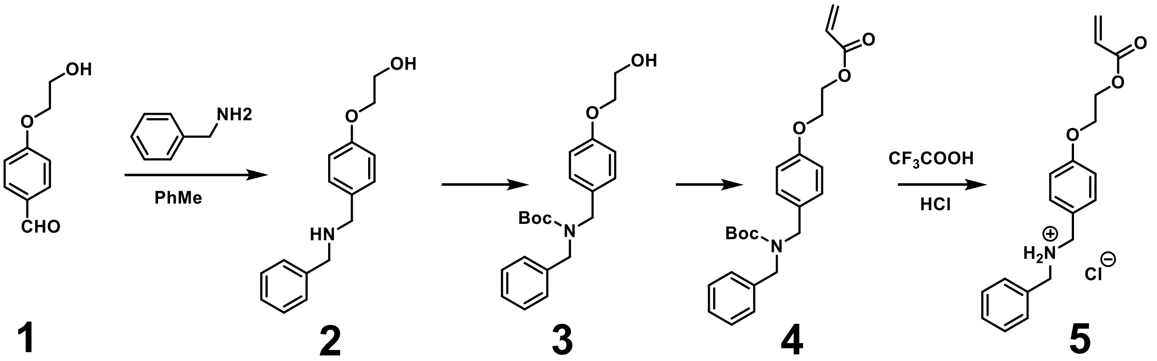

Fig. S1. Synthetic route to guest monomer **5**.

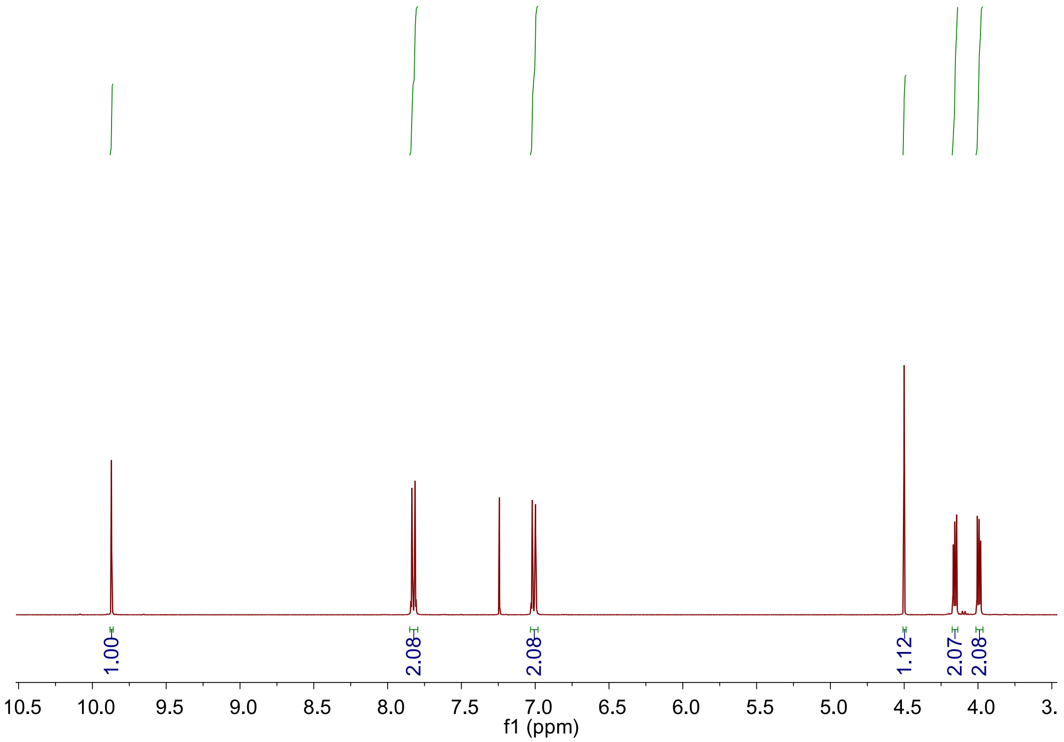

Fig. S2. ^{1}H NMR spectrum (400 MHz, $CDCl_3$, 298 K) of compound **1**.

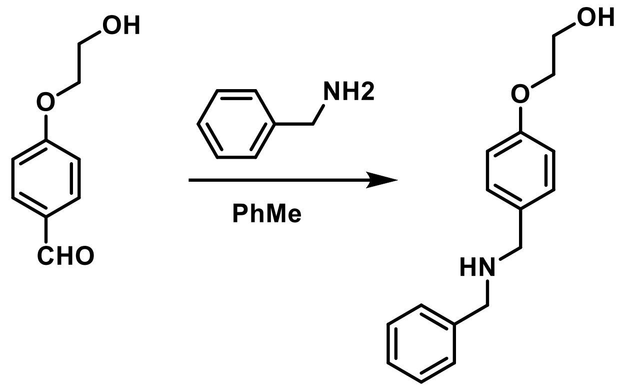

1 **2**

Preparation of 2: A solution of **1** (4.95 g, 0.024 mol) and benzylamine (2.59 g, 0.024 mol) in toluene (150 mL) was heated under reflux with stirring in a Dean-Stark apparatus for 14 h. After the reaction mixture was allowed to cool to room temperature, the solvent was removed in vacuo to give an intermediate imine as brownish solid. This solid was dissolved in hot MeOH (100 mL). $NaBH_4$ (2.73 g, 0.072 mol) was added portion-wise, and the mixture heated under reflux with stirring for 10 h. The reaction mixture was then allowed to cool to room temperature, and concentrated HCl was added (pH < 2). After evaporation of the volatiles, the residue was suspended in H_2O (50 mL) and extracted with CH_2Cl_2 (4 × 50 mL). The combined extracts were washed with 5% aqueous $NaHCO_3$ (2 × 60 mL) and H_2O (50 mL) and then dried ($MgSO_4$). Removal of the volatiles in vacuo and purification by silica gel column chromatography (eluent: $MeOH/CH_2Cl_2$, 1:30) afforded the amine **2** (5.74 g, 80%) as a pale-yellow solid. The ^1H NMR spectrum of compound **2** is shown in Fig. S3. ^1H NMR (400 MHz, $(CD_3)_2CO$, 298 K) δ (ppm): 7.38–7.33 (m, 2H), 7.31–7.24 (m, 4H), 7.21 (m, 1H), 6.89–6.84 (m, 2H), 4.05–4.00 (m, 2H), 3.86–3.81 (m, 2H), 3.74 (s, 2H), 3.69 (s, 2H). The ^{13}C NMR spectrum of **2** is shown in Fig. S4. ^{13}C NMR (101 MHz, $(CD_3)_2CO$) δ 158.02, 129.05, 127.97, 126.48, 114.13, 69.60, 60.55, 52.61, 52.11. HRESIMS of **2** is shown in Fig. S5: *m/z* calcd for $[M + H]^+$ $C_{16}H_{20}NO_2^+$, 258.1489; found 258.1493; error -1.59 ppm.

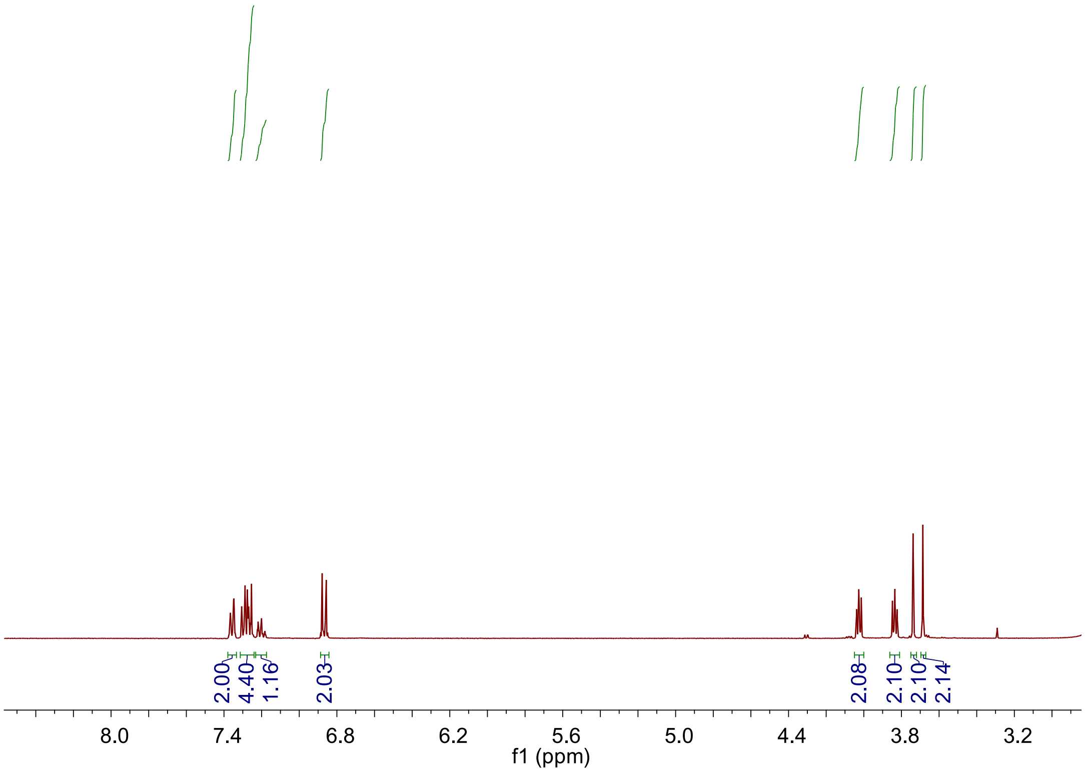

Fig. S3. ^{1}H NMR spectrum (400 MHz, $(CD_3)_2CO$, 298 K) of compound **2**.

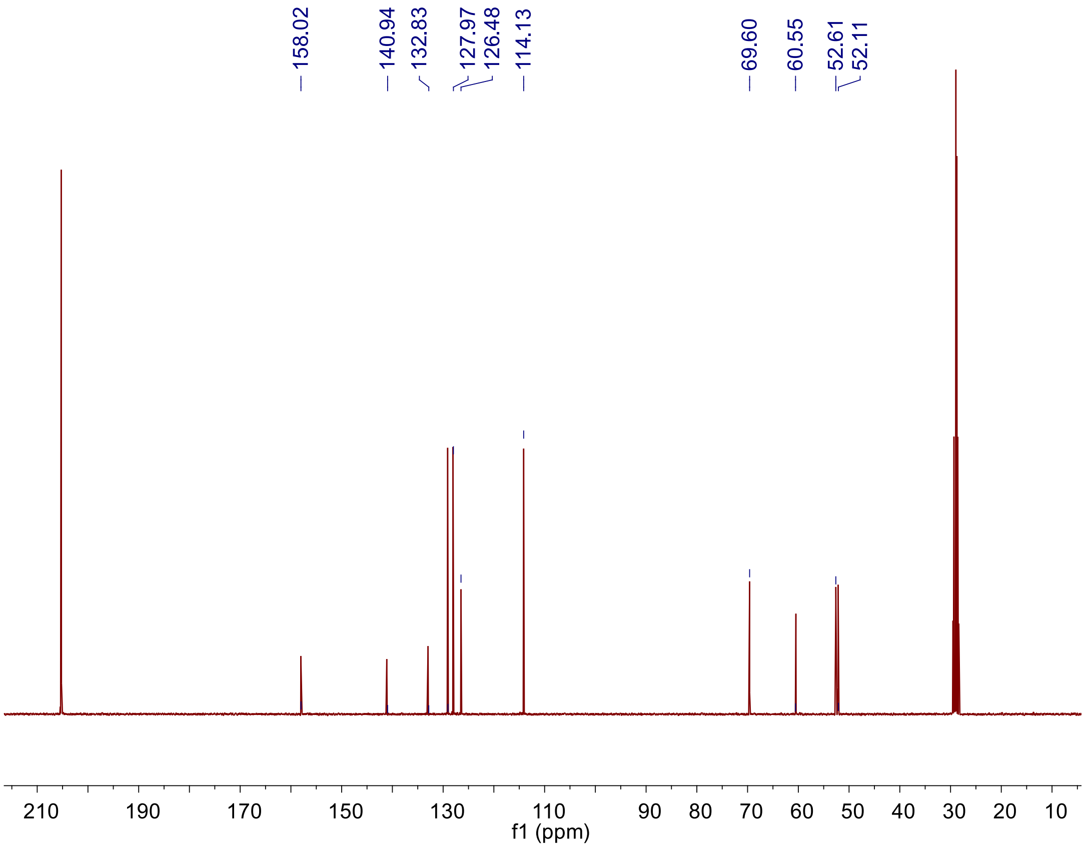

Fig. S4. ^{13}C NMR spectrum (400 MHz, $(CD_3)_2CO$, 298 K) of compound **2**.

Target Compound Screening Report

Results Acquired by The University of Texas at Austin Mass Spectrometry Facility

**Data File**	MSF21-0573(WH-xitingketi-1)_hrESIpos2.d	**Sample Name**	0573(WH-xitingketi-1)	**Comment**	0573(WH-xitingketi-1)
**Position**	P1-C3	**Instrument Name**	Instrument 1	**User Name**	
**Acq Method**	FIA_pos.m	**Acquired Time**	4/19/2021 12:51:59 PM	**DA Method**	KS.m

MS Zoomed Spectrum

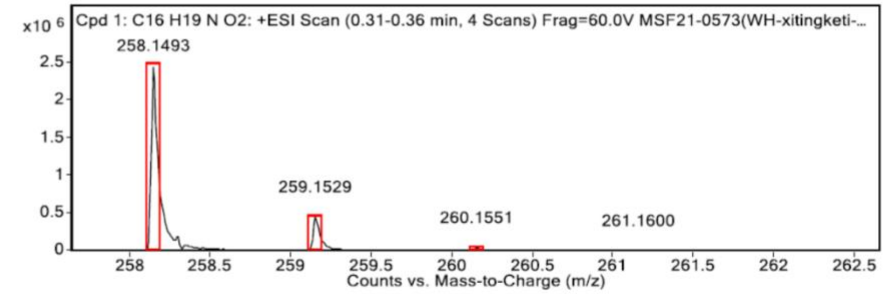

MS Spectrum Peak List

Obs. m/z	Calc. m/z	Charge	Abundance	Formula	Ion Species	Tgt Mass Error (ppm)
258.1493	258.1489	1	2487221	C16H19NO2	(M+H)+	-1.59
259.1529	259.1521	1	456102	C16H19NO2	(M+H)+	-3.12
260.1551	260.1549	1	45590	C16H19NO2	(M+H)+	-0.65
261.1600	261.1576	1	4322	C16H19NO2	(M+H)+	-9.47

--- End Of Report ---

Fig. S5. HRESIMS mass spectrum of compound **2**.

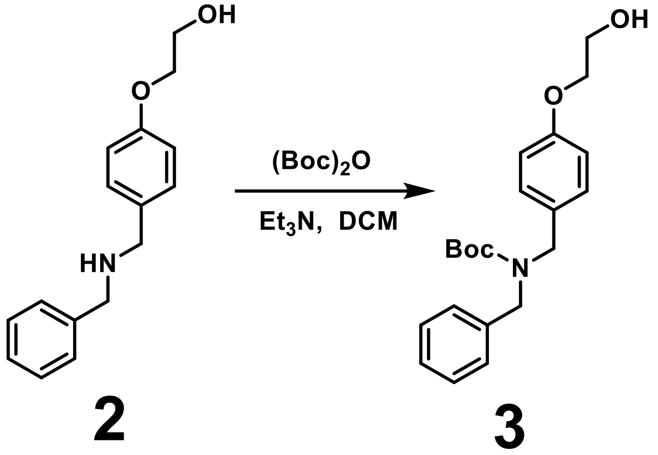

*Preparation of **3**:* To a solution of **2** (2.72 g, 13.9 mmol) and triethylamine (5.62 g, 31.0 mmol) in CH_2Cl_2 (DCM; 270 mL) was added di-*tert*-butyldicarbonate (3.79 g; 17.4 mmol) and the resulting solution was allowed to stir 18 h at rt before being concentrated under reduced pressure. The residue was partitioned between Et_2O/H_2O; the phases were separated, and the aqueous phase extracted twice more with Et_2O. The aqueous phase was brought to pH 4 with solid citric acid and extracted with $CHCl_3$ (3 x 100 mL). The organic extracts were combined, dried (Na_2SO_4) and concentrated under reduced pressure to afford 2.58 g (63%) **3** as a white solid. The ^{1}H NMR spectrum of **3** is shown in Fig. S6. ^{1}H NMR (400 MHz, $(CD_3)_2CO$, 298 K) δ (ppm): 7.32 (m, 2H), 7.25 (s, 3H), 7.18 (d, J = 7.1 Hz, 2H), 6.89 (d, J = 8.7 Hz, 2H), 4.29 (d, J = 27.9 Hz, 4H), 4.05–4.03 (m, 2H), 3.84 (d, J = 5.2 Hz, 2H), 3.75 (s, 1H), 1.45 (s, 9H). The ^{13}C NMR spectrum of **3** is shown in Fig. S7. ^{13}C NMR (101 MHz, $(CD_3)_2CO$, 298 K) δ (ppm) 158.45, 155.40, 130.27, 128.36, 126.85, 114.49, 79.12, 60.56, 27.66. HRESIMS of **3** is shown in Fig. S8: m/z calcd for $[M + Na]^+$ $[C_{21}H_{27}NO_4 + Na]^+$, 380.1832; found 380.1825; error 2.02 ppm.

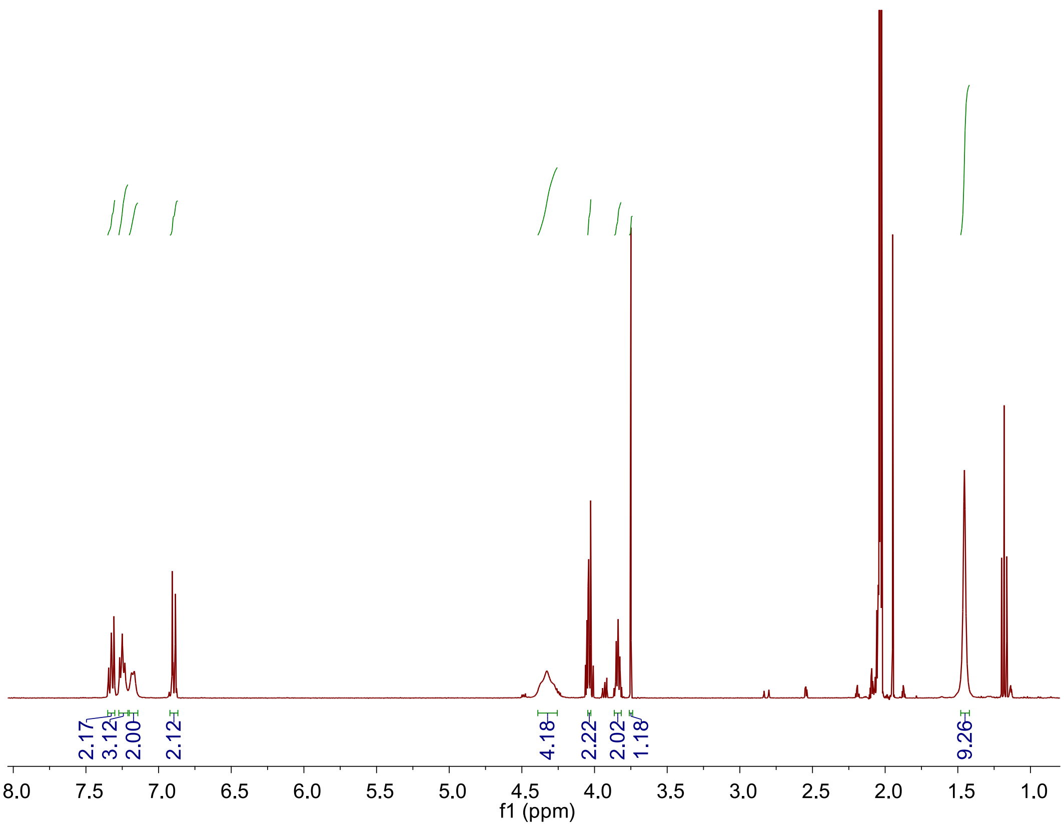

Fig. S6. ^{1}H NMR spectrum (400 MHz, $(CD_3)_2CO$, 298 K) of compound **3**.

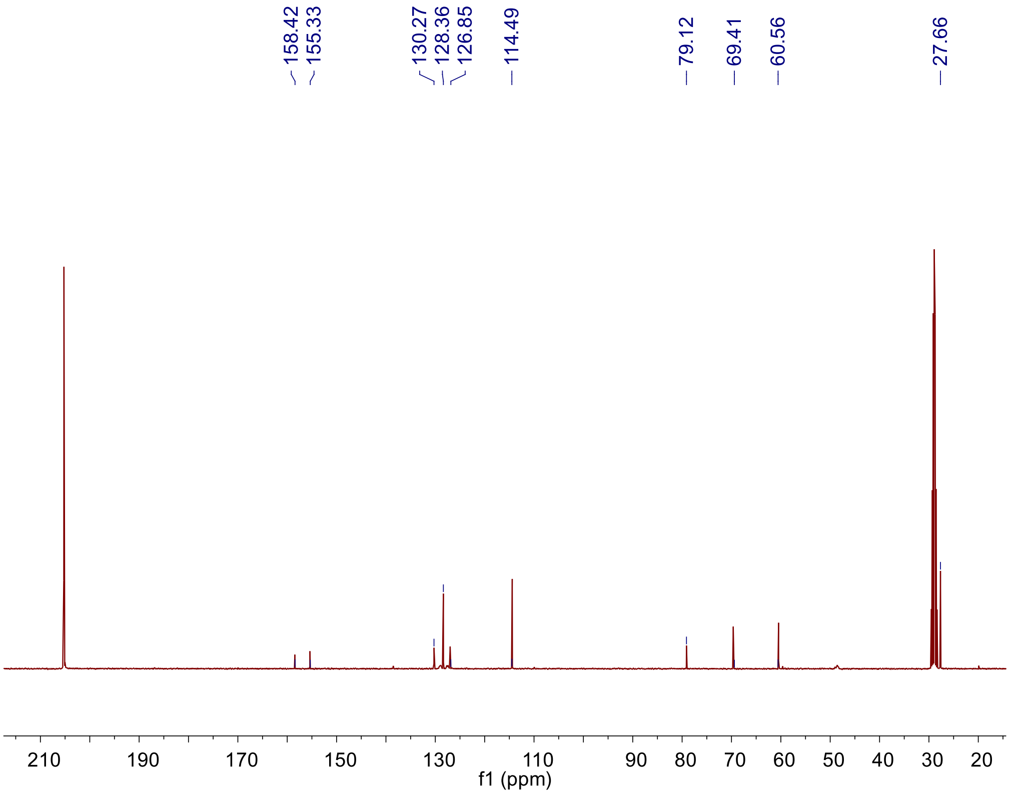

Fig. S7. ^{13}C NMR spectrum (400 MHz, $(CD_3)_2CO$, 298 K) of compound **3**.

Target Compound Screening Report

Results Acquired by The University of Texas at Austin Mass Spectrometry Facility

**Data File**	MSF21-0579(WH-xitingketi-2)_hrESIpos1.d	**Sample Name**	0579(WH-xitingketi-2)	**Comment**	0579(WH-xitingketi-2)
**Position**	P1-C2	**Instrument Name**	Instrument 1	**User Name**	
**Acq Method**	FIA_pos.m	**Acquired Time**	4/26/2021 1:22:00 PM	**DA Method**	KS.m

MS Zoomed Spectrum

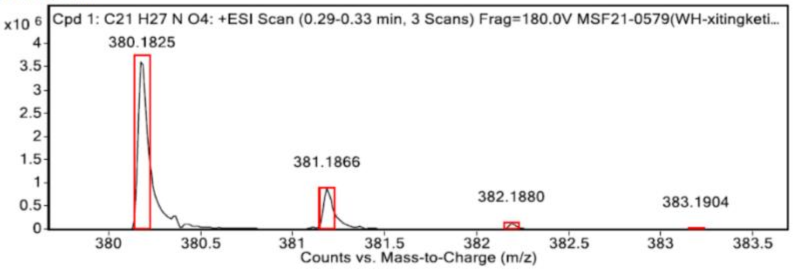

MS Spectrum Peak List

Obs. m/z	Calc. m/z	Charge	Abundance	Formula	Ion Species	Tgt Mass Error (ppm)
380.1825	380.1832	1	3737321	C21H27NO4	(M+Na)+	2.02
381.1866	381.1865	1	879898	C21H27NO4	(M+Na)+	-0.15
382.1880	382.1893	1	130575	C21H27NO4	(M+Na)+	3.25
383.1904	383.1919	1	15058	C21H27NO4	(M+Na)+	3.97
384.1883	384.1946	1	1888	C21H27NO4	(M+Na)+	16.3

--- End Of Report ---

Fig. S8. HRESIMS mass spectrum of compound **3**.

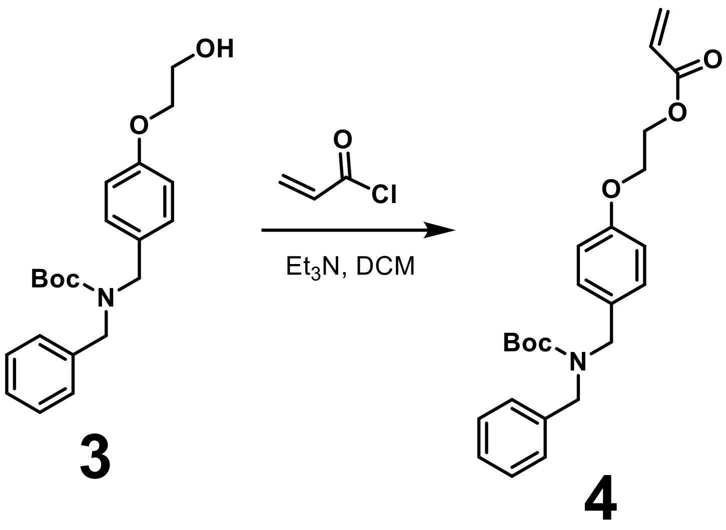

*Preparation of **4:*** Intermediate **3** (2.50 g, 0.007 mol) and triethylamine (1.42 g, 0.014 mol) were dissolved in 150 mL of dried DCM with the flask cooled in an ice bath. Acryloyl chloride (1.27 g, 0.014 mol) was added to the solution still on the ice bath. Then, the solution was stirred for 6 hours at room temperature. It was then concentrated under reduced pressure to give a white solid, which was purified by flash column chromatography (MeOH/DCM, 1:1 *v*/*v*) to afford **3** as a white solid (2.56 g, 89%). The ^{1}H NMR spectrum of **4** is shown in Fig. S9. ^{1}H NMR (400 MHz, $(CD_3)_2CO$, 298 K) δ (ppm): 7.36–7.30 (m, 2H), 7.28–7.16 (m, 5H), 6.93 (d, J = 8.6 Hz, 2H), 6.37 (m, 1H), 6.16 (m, 1H), 5.90 (m, 1H), 4.47 (d, J = 4.8 Hz, 2H), 4.30 (d, J = 22.2 Hz, 4H), 4.27 (t, J = 2.9 Hz, 2H), 1.45 (s, 9H). The ^{13}C NMR spectrum of **4** is shown in Fig. S10. ^{13}C NMR (101 MHz, $(CD_3)_2CO$) δ (ppm) 165.37, 157.99, 155.41, 130.67, 130.14, 128.79, 128.32, 126.99, 114.49, 79.13, 65.89, 62.77, 27.65. HRESIMS of **4** is shown in Fig. S11: *m*/*z* calcd for $[M + Na]^+$ $[C_{24}H_{29}NO_5 + Na]^+$, 434.1938; found 434.1933; error 1.12 ppm.

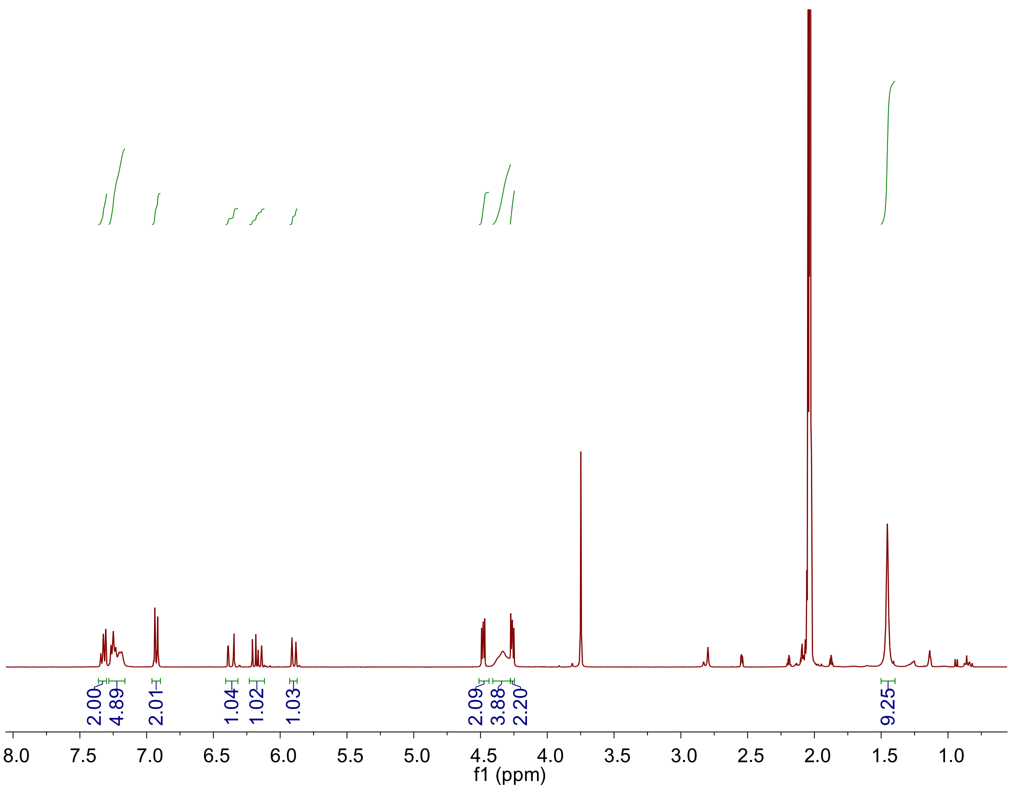

Fig. S9. ^{1}H NMR spectrum (400 MHz, $(CD_3)_2CO$, 298 K) of compound **4**.

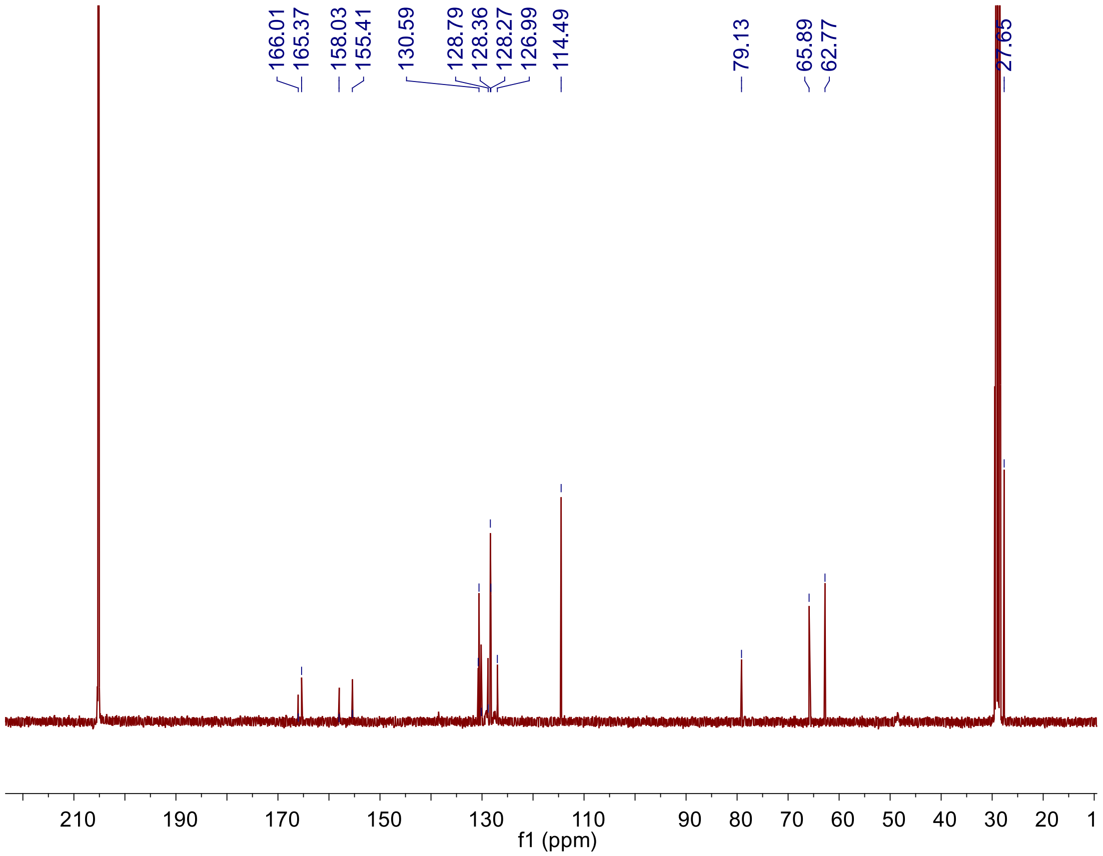

Fig. S10. ^{13}C NMR spectrum (400 MHz, $(CD_3)_2CO$, 298 K) of compound **4**.

Target Compound Screening Report

Results Acquired by The University of Texas at Austin Mass Spectrometry Facility

**Data File**	MSF21-0733(WH-xitingketi-3)_hrESIpos1.d	**Sample Name**	0733(WH-xitingketi-3)	**Comment**	0733(WH-xitingketi-3)
**Position**	P1-A8	**Instrument Name**	Instrument 1	**User Name**	
**Acq Method**	FIA_pos.m	**Acquired Time**	5/4/2021 12:18:11 PM	**DA Method**	KS.m

MS Zoomed Spectrum

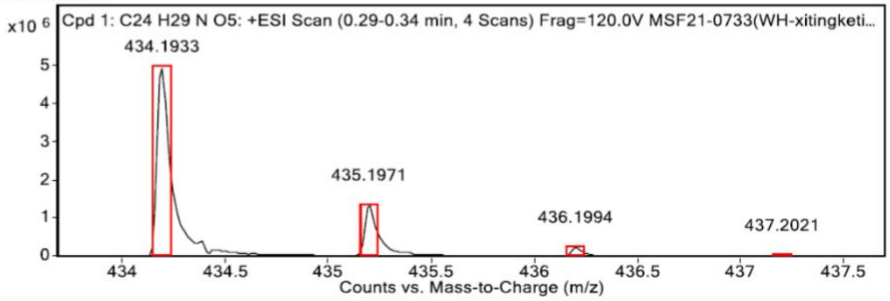

MS Spectrum Peak List

Obs. m/z	Calc. m/z	Charge	Abundance	Formula	Ion Species	Tgt Mass Error (ppm)
434.1933	434.1938	1	5000254	C24H29NO5	(M+Na)+	1.12
435.1971	435.1971	1	1347855	C24H29NO5	(M+Na)+	0.11
436.1994	436.1999	1	236951	C24H29NO5	(M+Na)+	1.09
437.2021	437.2026	1	24788	C24H29NO5	(M+Na)+	0.95
438.2021	438.2052	1	2504	C24H29NO5	(M+Na)+	7.03

--- End Of Report ---

Fig. S11. HRESIMS mass spectrum of compound **4**.

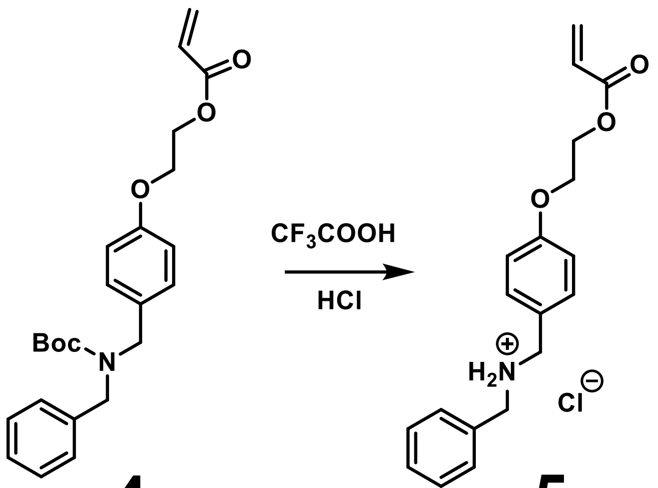

Preparation of ***5****:* Compound **4** (2.50 g, 0.006 mol) and trifluoroacetate (1.37 g, 0.012 mol) were dissolved in 150 mL of dry DCM. Then, the solution was stirred at room temperature for 5 hours, and then concentrated under reduced pressure to give a white solid. This solid was dissolved in 100 mL MeOH. HCl was added to the solution. The solution was then stirred at room temperature for 10 hours, and then concentrated under reduced pressure to give a white solid, which was recrystallized from acetonitrile to afford **5** as a white solid (2.08 g, 80%). The ^{1}H NMR spectrum of **5** is shown in Fig. S12. ^{1}H NMR (400 MHz, CD_3CN, 298 K) δ (ppm): 9.66 (s, 1H), 7.59 (m, 2H), 7.53 (d, J = 8.7 Hz, 2H), 7.43–7.37 (m, 3H), 6.93 (d, J = 8.7 Hz, 2H), 6.37 (m, 1H), 6.16 (m, 1H), 5.89 (m, 1H), 4.47–4.39 (m, 2H), 4.23–4.15 (m, 2H), 3.99 (d, J = 16.2 Hz, 4H). The ^{13}C NMR spectrum of **5** is shown in Fig. S13. ^{13}C NMR (400 MHz, CD_3CN, 298 K) δ (ppm): 165.74, 159.60, 132.08, 131.38, 131.03, 130.30, 129.16, 128.75, 128.05, 123.54, 114.62, 67.18, 63.52, 50.15, 49.85. HRESIMS of **5** is shown in Fig. S14: m/z calcd for M^+ $C_{19}H_{22}NO_3^+$, 312.1594; found 312.1594; error -0.05 ppm.

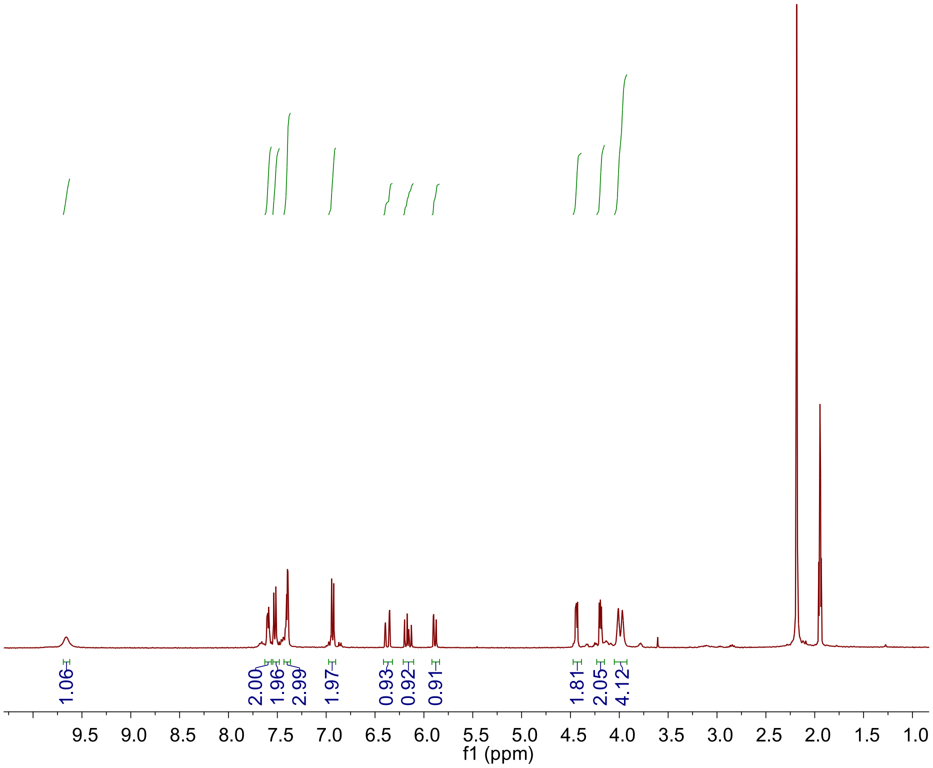

Fig. S12. ^{1}H NMR spectrum (400 MHz, CD_3CN, 298 K) of compound **5**.

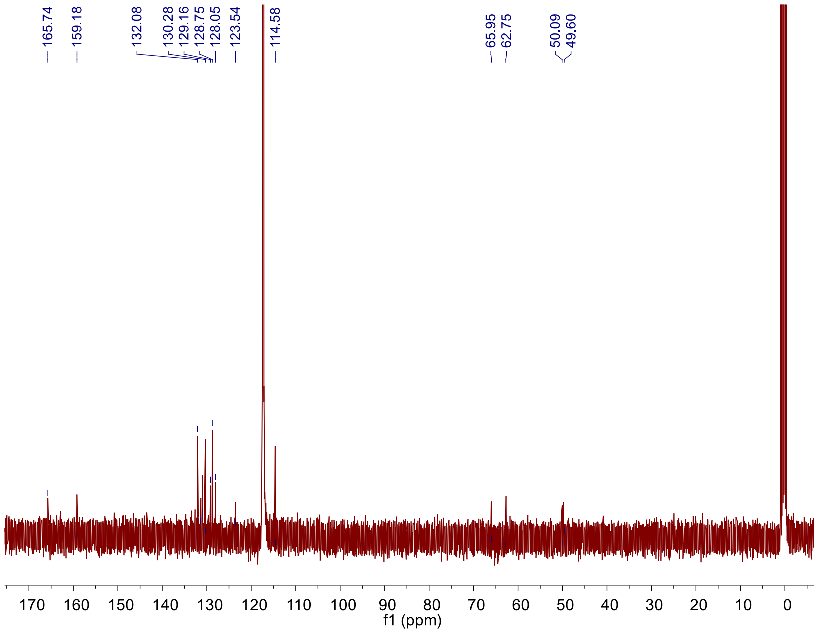

Fig. S13. ^{13}C NMR spectrum (400 MHz, CD_3CN, 298 K) of compound **5**.

Target Compound Screening Report

Results Acquired by The University of Texas at Austin Mass Spectrometry Facility

**Data File**	MSF21-0004(WH-xiting-keti)_hrESIpos1.d	**Sample Name**	0004(WH-xiting-keti)	**Comment**	0004(WH-xiting-keti)
**Position**	P1-A9	**Instrument Name**	Instrument 1	**User Name**	
**Acq Method**	FIA_pos.m	**Acquired Time**	1/6/2021 8:49:52 AM	**DA Method**	KS.m

MS Zoomed Spectrum

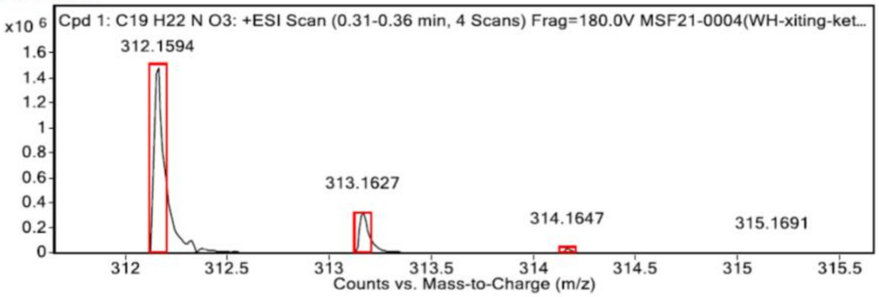

MS Spectrum Peak List

Obs. m/z	Calc. m/z	Charge	Abundance	Formula	Ion Species	Tgt Mass Error (ppm)
312.1594	312.1594	1	1514301	C19H22NO3	M+	-0.05
313.1627	313.1627	1	325532	C19H22NO3	M+	0.11
314.1647	314.1655	1	41249	C19H22NO3	M+	2.3
315.1691	315.1681	1	3041	C19H22NO3	M+	-3.18

--- End Of Report ---

Fig. S14. HRESIMS mass spectrum of compound **5**.

*3. Partial NOESY NMR spectrum (400 MHz, 298 K) of a CD_3CN solution 15.0 mM in **H** and 15.0 mM in **G**.*

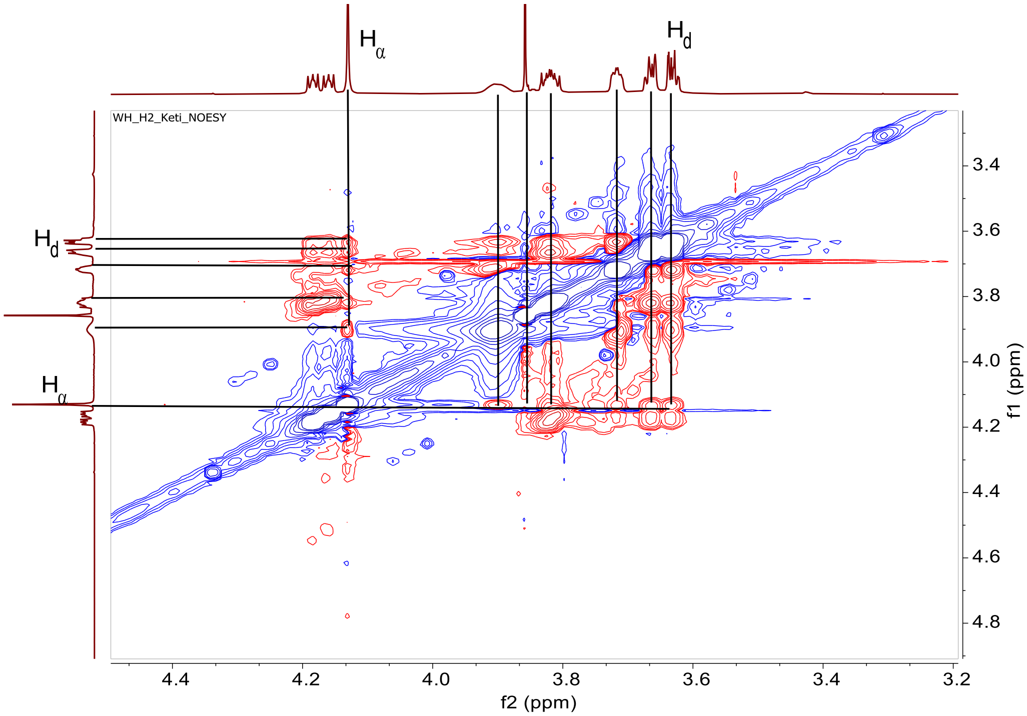

Fig. S15. Partial NOESY NMR spectrum (400 MHz, 298 K) of a CD_3CN solution of **H** and **G** (both at 15.0 mM**)**.

4. Determination of the association constant for ***H⊃G***

To determine the stoichiometry and association constant corresponding to the presumed interaction between **H** (**Host**) and **G** (**Guest**), ^{1}H NMR spectroscopic titrations were carried out using solutions that had a constant concentration of **Host** (5.00 mM) and varying concentrations of **Guest**. Using a non-linear curve-fitting method, the association constant corresponding to the interaction between guest **Guest** and **Host** was calculated. While not a proof, a 1:1 stoichiometry was inferred on the basis of a mole ratio (Job) plot.

The non-linear curve-fitting was based on the following equation:[S4]

$$\Delta\delta = (\Delta\delta_\infty/[G]_0)\,(0.5[H]_0 + 0.5([G]_0+1/K_a) - (0.5\,([H]_0{}^2+(2[H]_0(1/K_a - [G]_0)) + (1/K_a + [G]_0)^2)^{0.5})) \quad \text{(Eq. S1)}$$

Where $\Delta\delta$ is the chemical shift change of H_f/H_ζ on **Host** at $[G]_0$, $\Delta\delta_\infty$ is the chemical shift change of H_f/H_ζ when the host is completely complexed, $[H]_0$ is the fixed initial concentration of **Host**, and $[G]_0$ is the varying concentration of **Guest**.

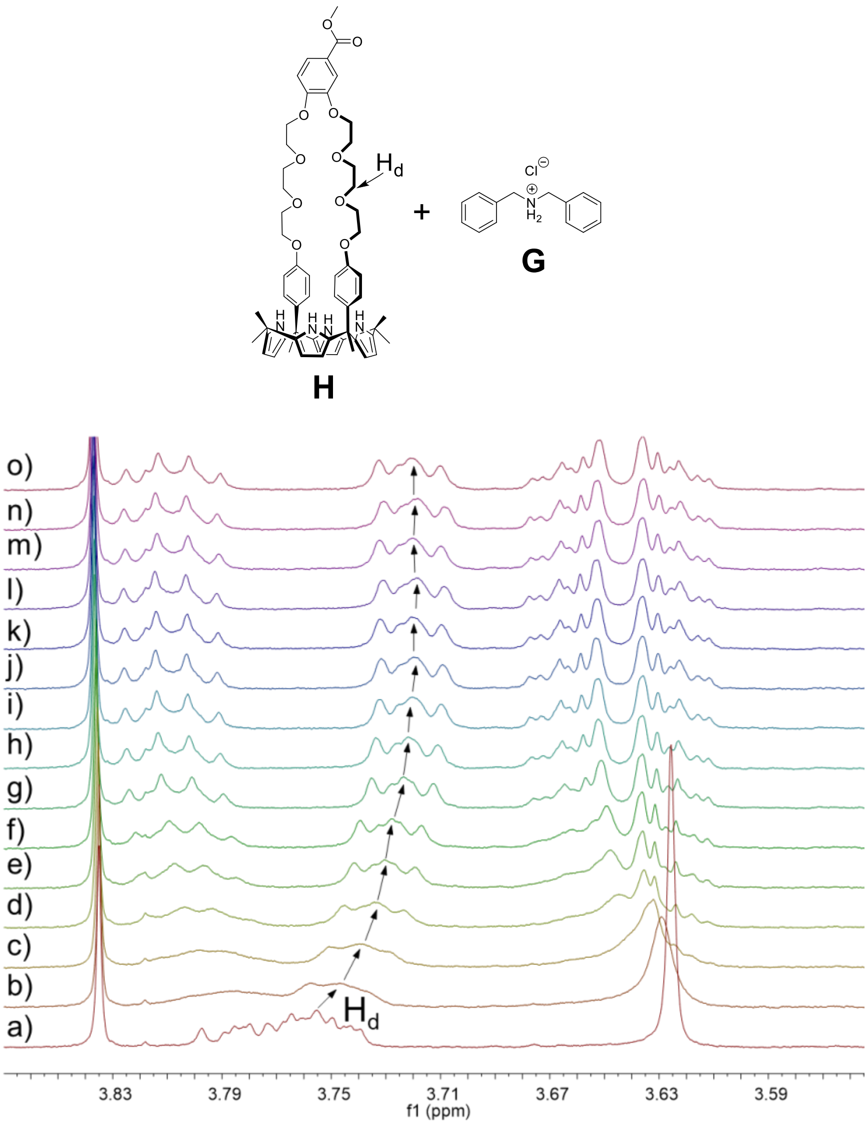

Fig. S16. ^{1}H NMR spectra (400 MHz, CD_3CN, 298 K) of **H** (**Host**) recorded at a concentration of 5.00 mM in the presence of different concentrations of **G** (**Guest**): (a) 0.00 mM; (b) 1.18 mM; (c) 2.23 mM; (d) 3.18 mM; (e) 4.03 mM; (f) 4.8 mM; (g) 6.94 mM; (h) 8.58 mM; (i) 9.87 mM; (j) 10.91 mM; (k) 11.77 mM; (l) 12.5 mM; (m) 13.65 mM; (n) 14.52 mM; (o) 15.2 mM.

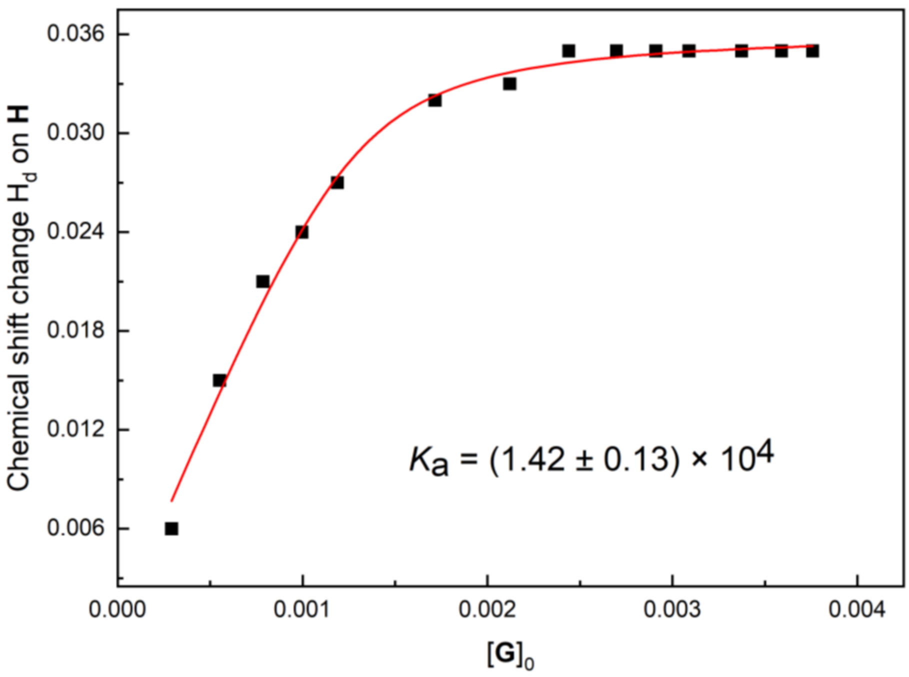

Fig. S17. Changes in the chemical shift corresponding to H_d on **H** (**Host**) as a function of added **G** (**Guest**). The red solid line was obtained from a non-linear curve-fitting using Eq. S1.

*5. NMR spectral analysis the 1:1 mixture of **H** and **G** recorded in the absence and presence of added NaCl, KCl, $MgCl_2$ and $CaCl_2$*

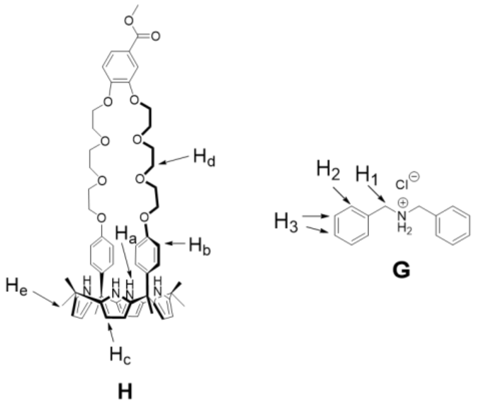

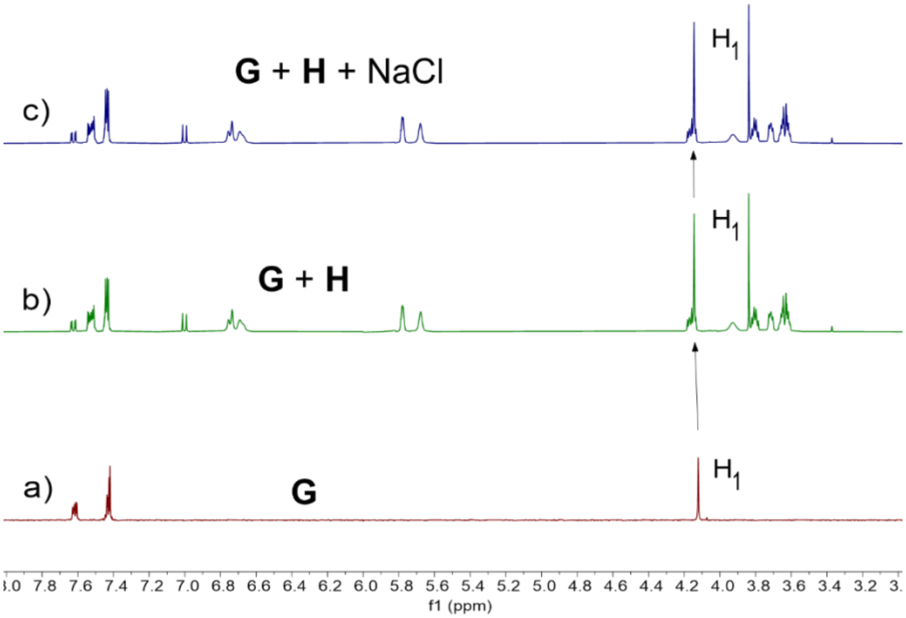

Fig. S18. Partial ^{1}H NMR spectra (400 MHz, CD_3CN, 295 K): a) **G** (5.00 mM); b) **G** (5.00 mM) + **H** (5.00 mM); c) after addition of 0.29 g/L NaCl to b.

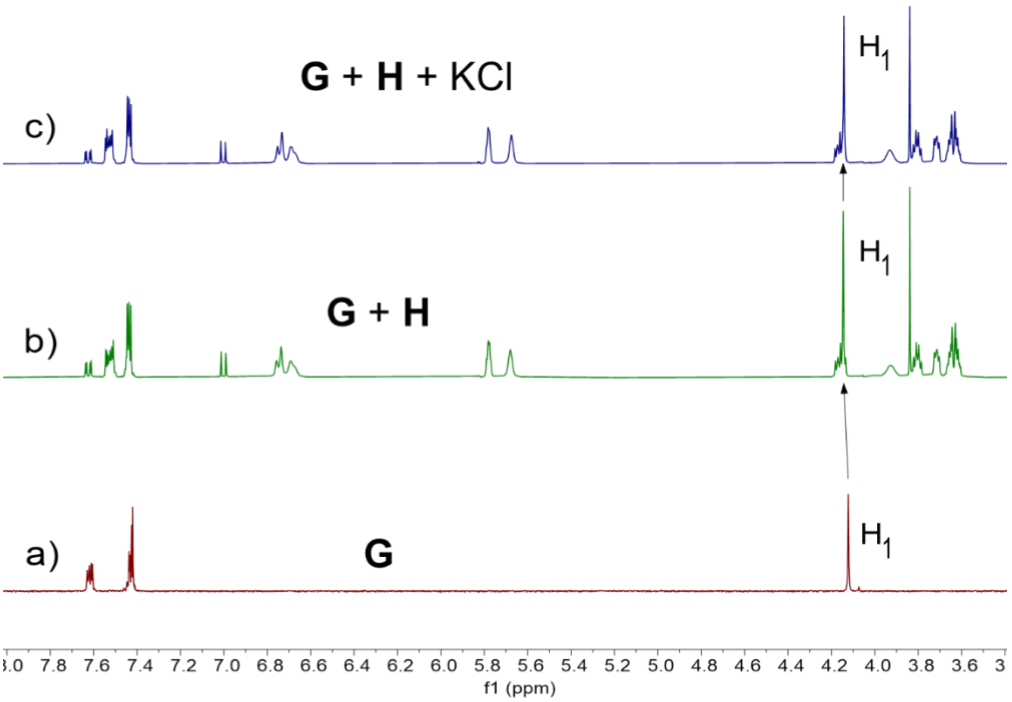

Fig. S19. Partial ^{1}H NMR spectra (400 MHz, CD_3CN, 295 K): a) **G** (5.00 mM); b) **G** (5.00 mM) + **H** (5.00 mM); c) after addition of 0.37 g/L KCl to b.

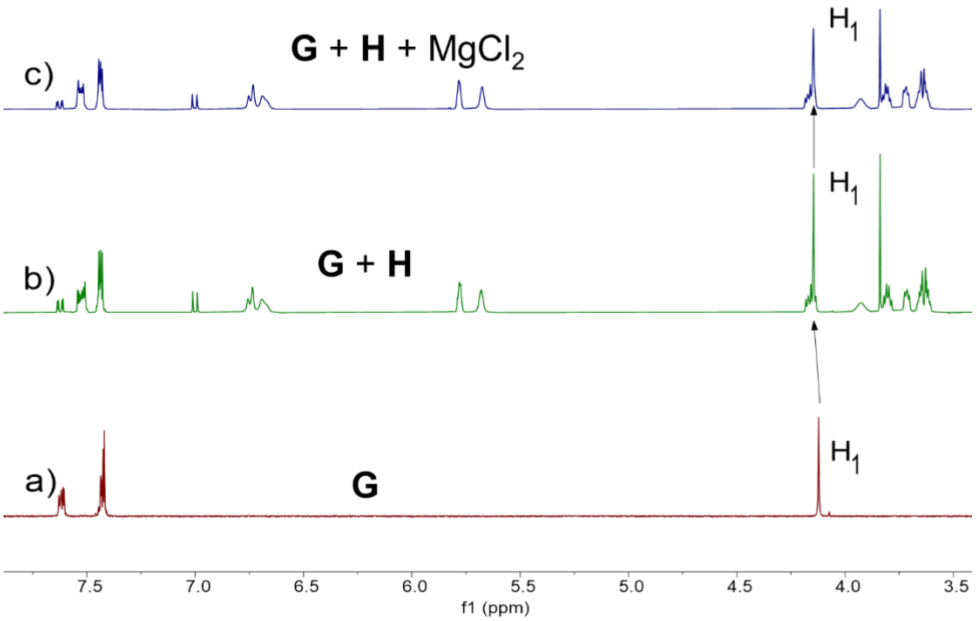

Fig. S20. Partial ^{1}H NMR spectra (400 MHz, CD_3CN, 295 K): a) **G** (5.00 mM); b) **G** (5.00 mM) + **H** (5.00 mM); c) after addition of 0.48 g/L $MgCl_2$ to b.

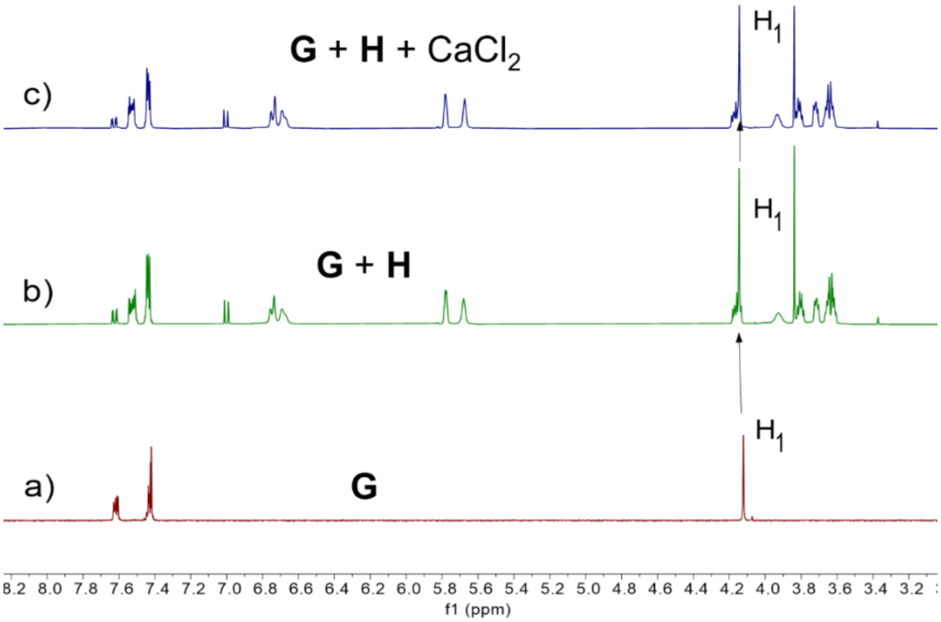

Fig. S21. Partial ^{1}H NMR spectra (400 MHz, CD_3CN, 295 K): a) **G** (5.00 mM); b) **G** (5.00 mM) + **H** (5.00 mM); c) after addition of 0.55 g/L $CaCl_2$ to b.

6. Synthesis of polymers ***P1*** *and* ***P2***

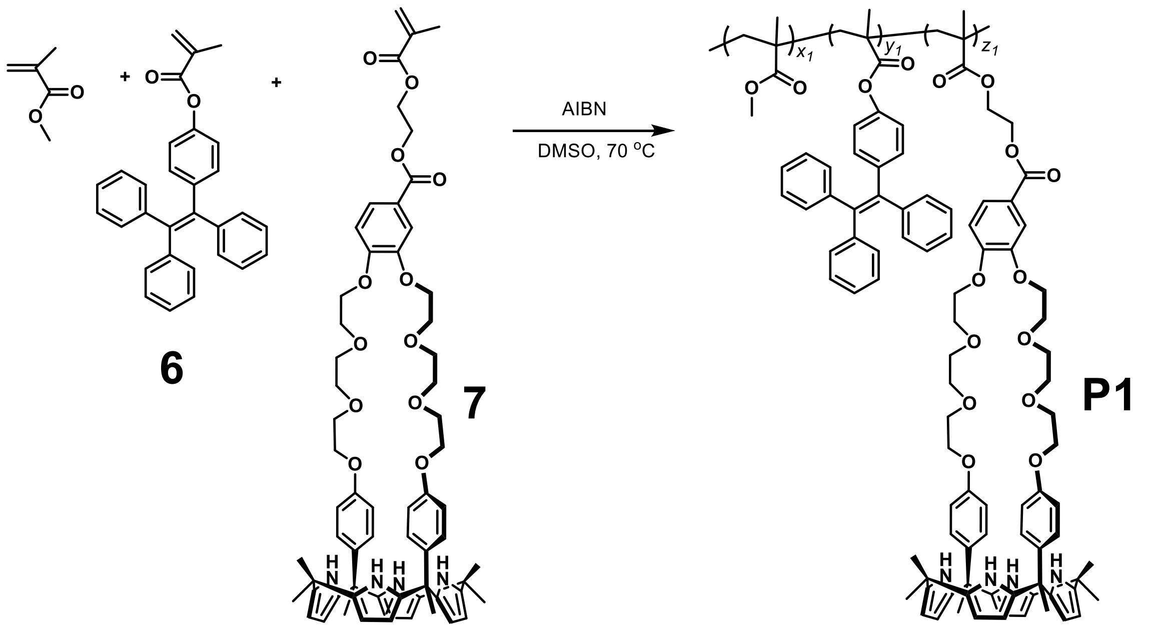

Polymer **P1** was prepared from compounds **6**, **7**, and methyl methacrylate by free radical polymerization. A mixture of compound **6** (193 mg, 0.46 mmol), compound **7** (500 mg, 0.46 mmol), and methyl methacrylate (0.50 mL, 4.6 mmol) in 10 mL of dimethyl sulfoxide (DMSO) was stirred at room temperature. A stream of argon (Ar) was bubbled through for 30 min. Azobisisobutyronitrile (AIBN; 1.60 mg, 0.010 mmol) was added in one portion. The mixture was stirred for 10 min, sealed with a rubber septum and heated to 70 °C for 24 h. Polymerization was quenched by rapid freezing in liquid nitrogen. The solution was dropped into 500 mL of methanol, and the precipitated solid was collected by vacuum filtration. The precipitate was dissolved in THF and then dropped into methanol to obtain the precipitate again, which was repeated three times. The collected polymer was dried in vacuum; yield 705 mg (61%).

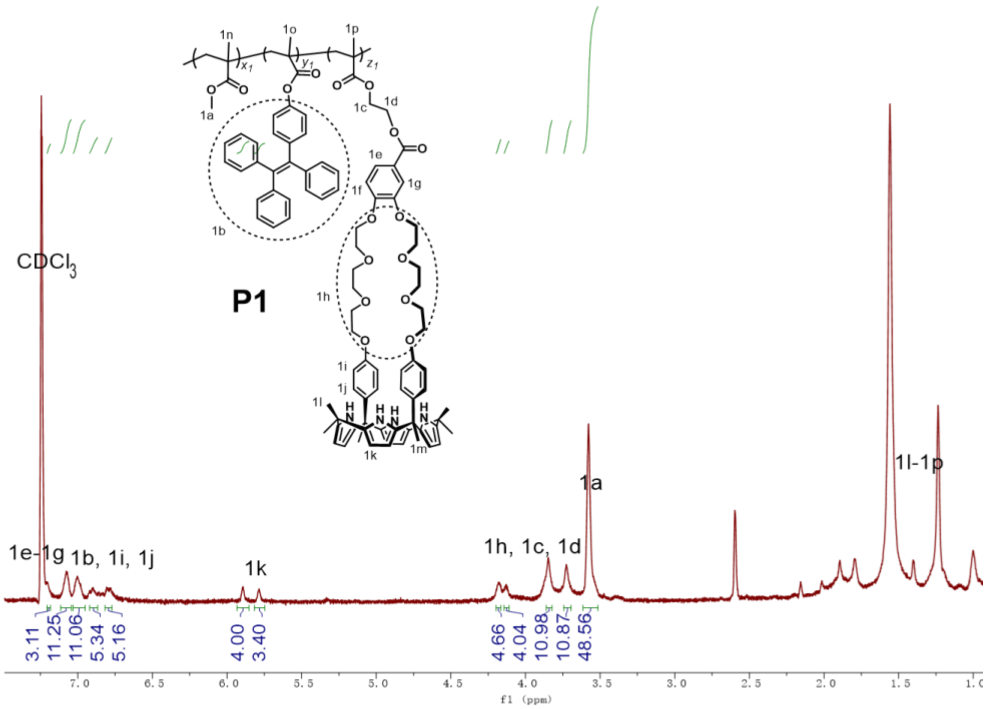

Fig. S22. ^{1}H NMR spectrum (400 MHz, $CDCl_3$, 298 K) of polymer **P1**.

The ratio of $x_1/y_1/z_1$ was 16.19/1.31/1, for polymer **P1**, as calculated based on the integrations of the peaks of H_{1a}, H_{1b}, and H_{1k} in Fig.. S22.

Table S1. GPC analysis of polymer **P1** using conventional calculations, with polystyrene as the standard and DMF as the solvent.

M_n	M_w	M_z	PDI
30554	47640	69274	1.559

According to M_n and the ratio of $x_1/y_1/z_1$, the values of x_1, y_1, and z_1 were calculated to be 153, 12.3 and 9.4, respectively.

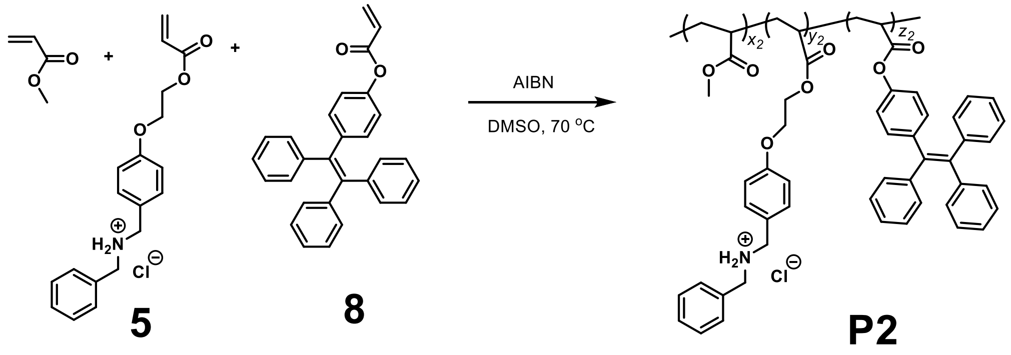

Polymer **P2** was prepared from compounds **5**, **8**, and methyl acrylate by free radical polymerization. A mixture of compound **5** (174 mg, 0.50 mmol), compound **8** (201 mg, 0.50 mmol), and methyl methacrylate (0.45 mL, 5.0 mmol) in 10 mL of dimethyl sulfoxide (DMSO) was stirred at room temperature. A stream of argon (Ar) was bubbled through for 30 min. AIBN (1.64 mg, 0.010 mmol) was added in one portion. The mixture was stirred for 10 min, sealed with a rubber septum and heated to 70 °C for 24 h. The polymerization was quenched by rapid freezing in liquid nitrogen. The solution was dropped into 500 mL of methanol, and the precipitated solid was collected by vacuum filtration. The precipitate was dissolved in THF and then dropped into methanol to obtain the precipitate again, which was repeated three times. The collected polymer was dried in vacuum; yield 600 mg (52%).

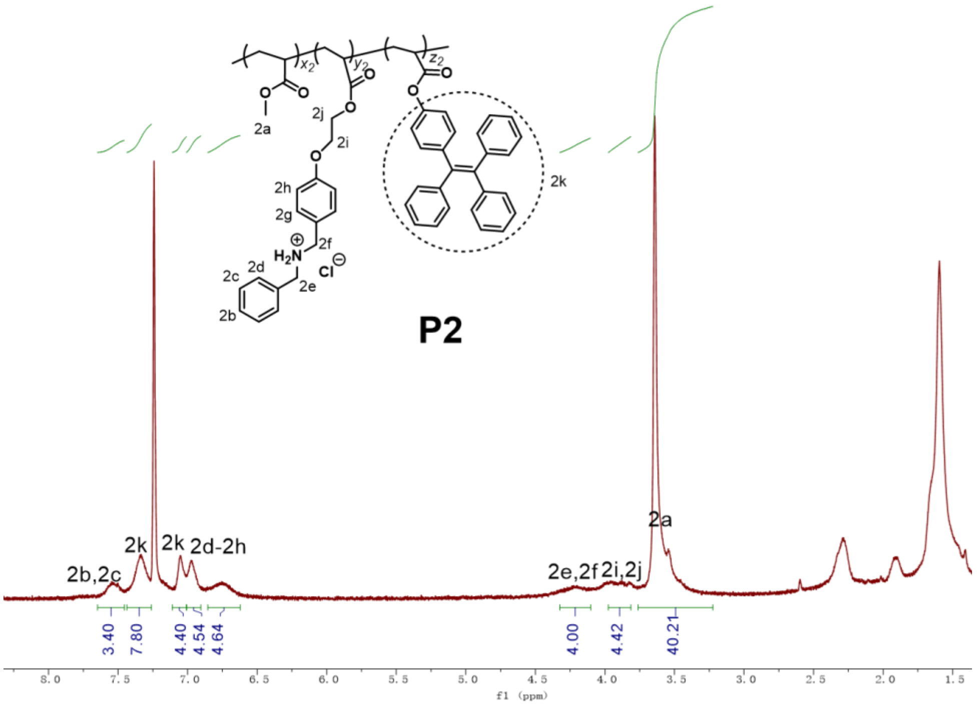

Fig. S23. ^{1}H NMR spectrum (400 MHz, $CDCl_3$, 298 K) of polymer **P2**.

The ratio of $x_2/y_2/z_2$ was 13.4/1/0.83, for polymer **P2**, as calculated based on the integrations of the peaks of H_{2a}, H_{2e}, H_{2f} and H_{2k} in Fig.. S23.

Table S2. GPC analysis of polymer **P2** using conventional calculations, with polystyrene as the standard and DMF as the solvent.

M_n	M_w	M_z	PDI
27186	43708	64710	1.608

According to M_n and the ratio of $x_2/y_2/z_2$, the values of x_2, y_2, and z_2 were calculated to be 198.6, 14.8 and 12.7, respectively.

7. DLS results and TEM images

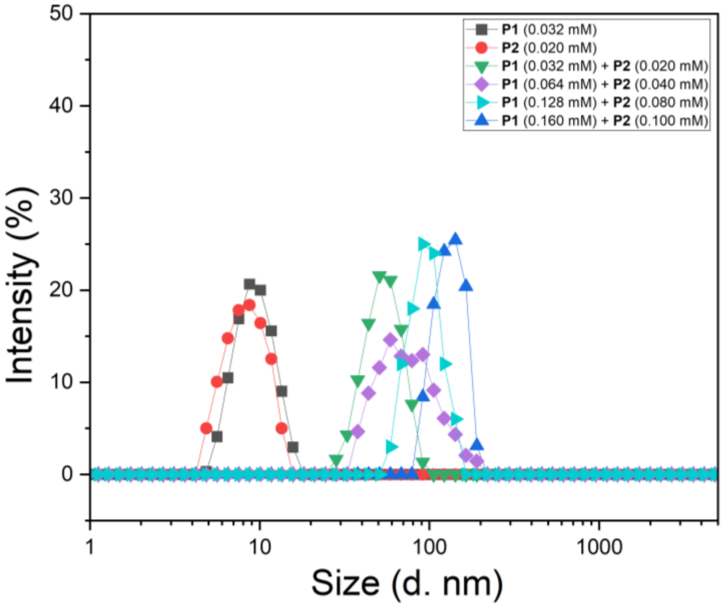

Fig. S24. DLS results of **P1** (0.032 mM), **P2** (0.020 mM) and **P1** + **P2** at different concentrations.

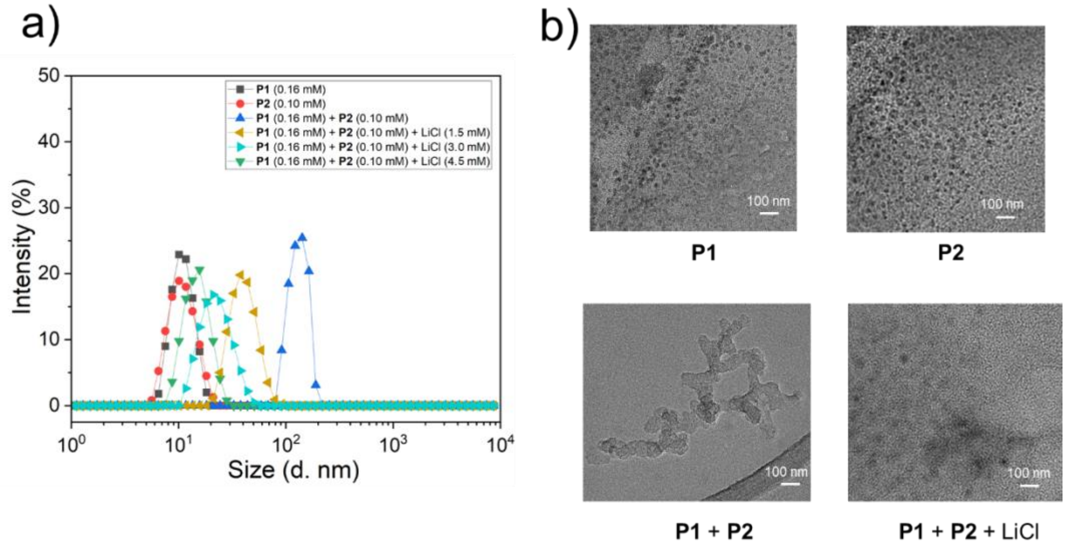

Fig. S25. (a) DLS results of **P1** (0.16 mM), **P2** (0.10 mM), **P1** (0.16 mM) + **P2** (0.10 mM), **P1** (0.16 mM) + **P2** (0.10 mM) + LiCl (1.5 mM), **P1** (0.16 mM) + **P2** (0.10 mM) + LiCl (3.0 mM), **P1** (0.16 mM) + **P2** (0.10 mM) + LiCl (4.5 mM); (b) TEM images of **P1** (0.16 mM), **P2** (0.10 mM), **P1** (0.16 mM) + **P2** (0.10 mM) and **P1** (0.16 mM) + **P2** (0.10 mM) + LiCl (4.5 mM).

*8. Linear correlation between the fluorescence emission intensity at 485 nm of **P1** + **P2** and LiCl concentration*

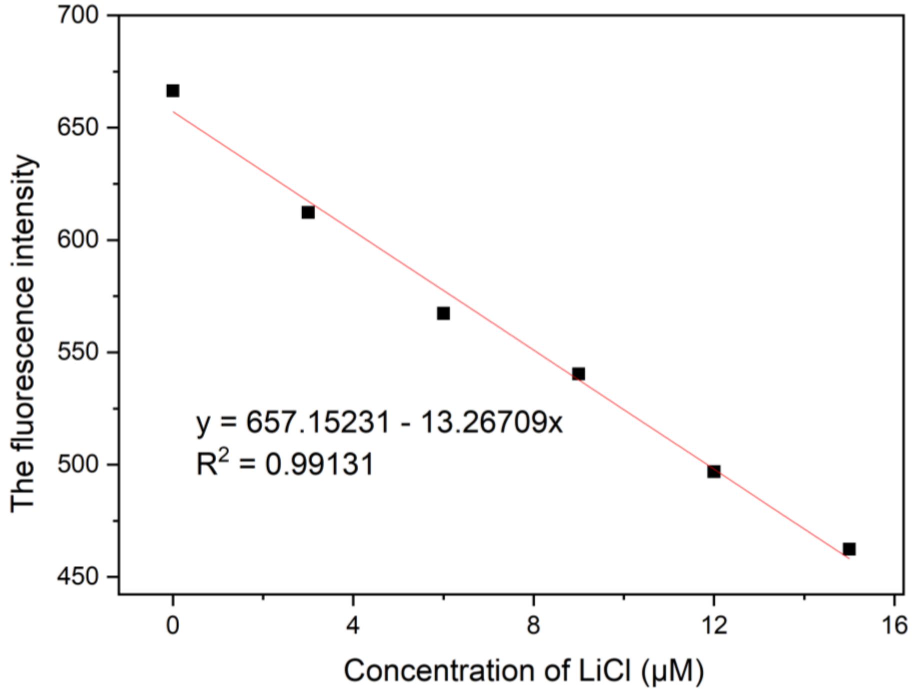

Fig. S26. Linear correlation between the fluorescence emission intensity at 485 nm of **P1** (1.60 μM) + **P2** (1.00 μM) and LiCl concentration (0–15 μM).

*9. Fluorescence spectra of **P1** (1.60 μM) + **P2** (1.00 μM) in acetonitrile with time after adding excess solid LiCl, NaCl, KCl, $MgCl_2$, and $CaCl_2$*

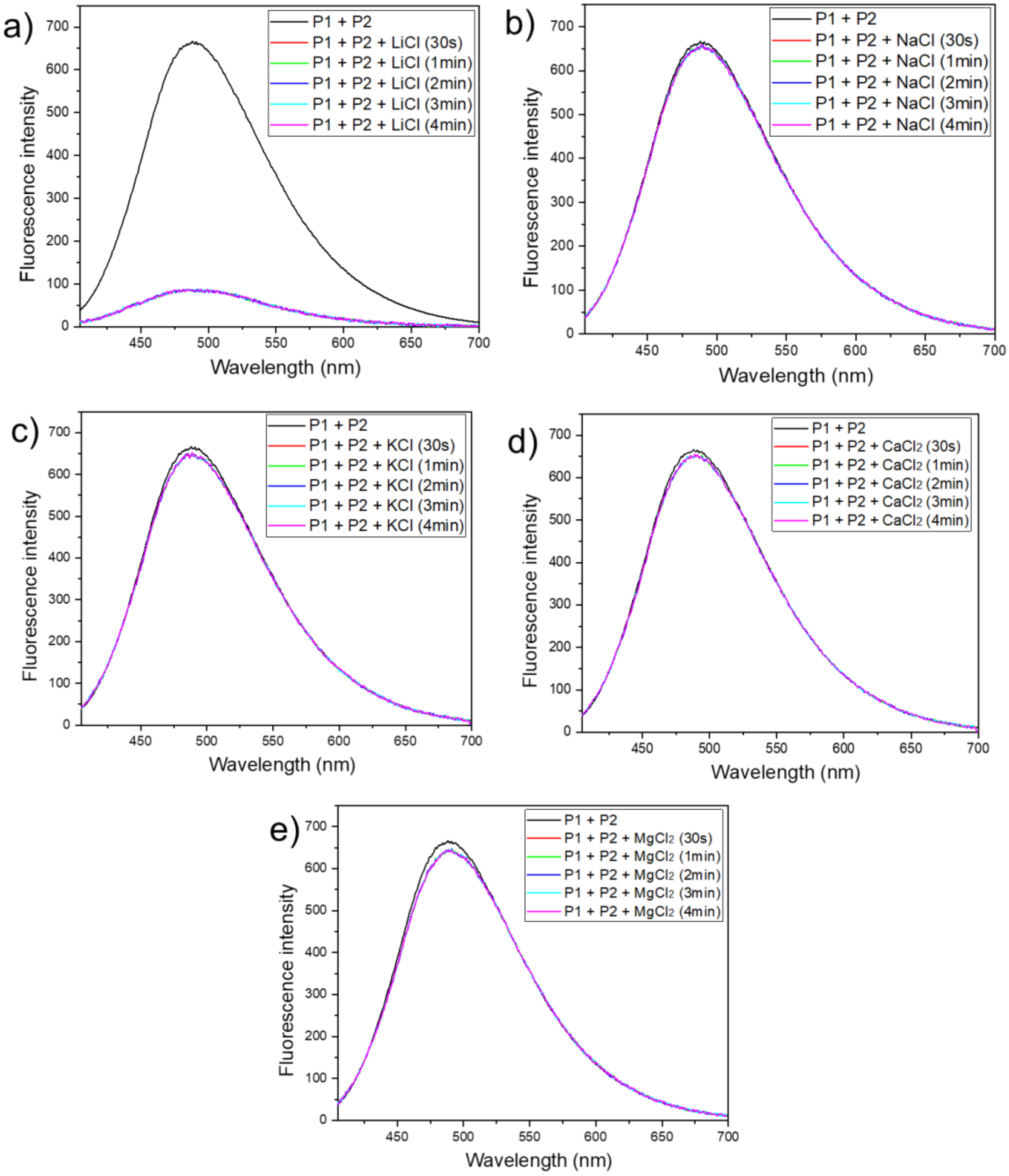

Fig. S27. Fluorescence spectra of **P1** (1.60 μM) + **P2** (1.00 μM) in acetonitrile with time after adding excess solid (a) LiCl, (b) NaCl, (c) KCl, (d) $MgCl_2$, and (e) $CaCl_2$. (The solubilities of LiCl, NaCl, KCl, $CaCl_2$ and $MgCl_2$ in acetonitrile are: 0.14, 0.0003, 0.0024 , 0.0002, 0.0285 (g salt/100 g of acetonitrile at 25 °C) respectively.)

*10. Fluorescence spectra of **P1** (1.60 μM) + **P2** (1.00 μM) in the competitive binding experiment*

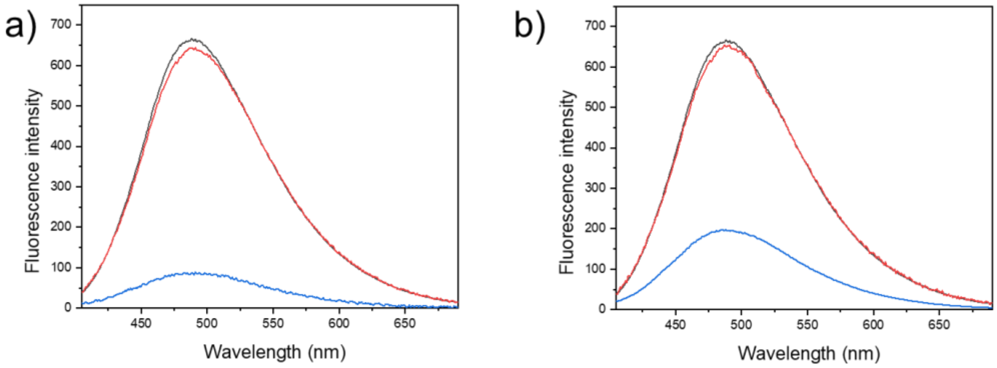

Fig. S28. (a) Fluorescence spectra of **P1** (1.60 μM) + **P2** (1.00 μM) in acetonitrile after adding excess solid NaCl, KCl, $MgCl_2$, and $CaCl_2$ mixture (red line) and then adding excess solid LiCl (blue line). (b) Fluorescence spectra of **P1** (1.60 μM) + **P2** (1.00 μM) in acetonitrile after adding 14.2 μM NaCl, KCl, $MgCl_2$, and $CaCl_2$ mixture (red line) and then adding 14.2 μM LiCl (blue line).

11. The instrument parameter conditions of the fluorescence test

Scan Software Version: 1.2(147)

Parameter List :

Instrument	Cary Eclipse
Instrument Serial Number	MY15130003
Data mode	Fluorescence
Scan mode	Emission
X Mode	Wavelength (nm)
Start (nm)	395.00
Stop (nm)	700.00
Ex. Wavelength (nm)	380.00
Ex. Slit (nm)	10
Em. Slit (nm)	10
Scan rate (nm/min)	600.00
Data interval (nm)	1.0000
Averaging Time (s)	0.1000
Excitation filter	Auto
Emission filter	Open
PMT voltage (V)	Medium
Corrected spectra	OFF

Fig. S29. *The instrument parameter conditions of the fluorescence test.*

12. X-ray experimental

X-ray experimental for the complex **H·G** (CCDC deposition number: 2183452)

Crystals grew as clusters of colorless prisms by slow evaporation from acetonitrile. The data crystal was separated from a cluster of crystals and had approximate dimensions; 0.24 x 0.13 x 0.032 mm. The data were collected on an Agilent Technologies SuperNova Dual Source diffractometer using a μ-focus Cu Kα radiation source (λ = 1.5418 Å) with collimating mirror monochromators. A total of 1042 frames of data were collected using ω-scans with a scan range of 1° and a counting time of 13.5 seconds per frame for frames collected with a detector offset of +/- 41.6° and 40.5 seconds per frame with frames collected with a detector offset of 107.1°. The data were collected at 100 K using an Oxford Cryostream low temperature device. Details of crystal data, data collection and structure refinement are listed in Table S3. Data collection, unit cell refinement and data reduction were performed using Rigaku Oxford Diffraction's CrysAlisPro V 1.171.41.93a.[S3] The structure was solved by direct methods using SHELXT[S4] and refined by full-matrix least-squares on F^2 with anisotropic displacement parameters for the non-H atoms using SHELXL-2018/3.[S5] Structure analysis was aided by use of the programs PLATON[S6] and OLEX2.[S7] The hydrogen atoms on the carbon atoms were calculated in ideal positions with isotropic displacement parameters set to 1.2xUeq of the attached atom (1.5xUeq for methyl hydrogen atoms).

The crystal was twinned. The twin law was determined using the CrysAlisPro software. The methyl ester group attached to the phenyl ring of the calixpyrrole cryptand was disordered about two positions. Refining the site occupancy factors resulted in a 77/23 mix of the two groups. Two fully occupied molecules of acetonitrile were also disordered. The function, $\Sigma w(|F_o|^2 - |F_c|^2)^2$, was minimized, where $w = 1/[(\sigma(F_o))^2 + (0.1123*P)^2]$ and $P = (|F_o|^2 + 2|F_c|^2)/3$. $R_w(F^2)$ refined to 0.192, with R(F) equal to 0.0589 and a goodness of fit, S, = 1.06. Definitions used for calculating R(F), $R_w(F^2)$ and the goodness of fit, S, are given below.[S8] The data were checked for secondary extinction effects but no correction was necessary. Neutral atom scattering factors and values used to calculate the linear absorption coefficient are from the International Tables for X-ray

Crystallography (1992).[S9] All Figures were generated using SHELXTL/PC.[S10] Tables of positional and thermal parameters, bond lengths and angles, torsion angles and Figures are included in the cif files, which may be obtained from the Cambridge Crystallographic Data Centre by referencing CCDC deposition number 2183452.

Table S3. Crystal data and structure refinement for **H·G**

Empirical formula	C76.46 H92.69 Cl N7.23 O11	
Formula weight	1324.45	
Temperature	100.03(16) K	
Wavelength	1.54184 Å	
Crystal system	triclinic	
Space group	P -1	
Unit cell dimensions	a = 12.7225(8) Å	α= 104.805(6)°.
	b = 15.8452(10) Å	β= 97.924(5)°.
	c = 18.7186(13) Å	γ = 96.664(5)°.
Volume	3568.0(4) $Å^3$	
Z	2	
Density (calculated)	1.233 Mg/m^3	
Absorption coefficient	0.996 mm^{-1}	
F(000)	1414	
Crystal size	0.244 x 0.129 x 0.032 mm^3	
Theta range for data collection	2.921 to 73.407°.	
Index ranges	-15<=h<=15, -19<=k<=19, -23<=l<=21	
Reflections collected	18197	
Independent reflections	18197 [R(int) = ?]	
Completeness to theta = 67.684°	98.7 %	
Absorption correction	Analytical	
Max. and min. transmission	0.971 and 0.867	
Refinement method	Full-matrix least-squares on F^2	
Data / restraints / parameters	18197 / 634 / 950	
Goodness-of-fit on F^2	1.042	
Final R indices [I>2sigma(I)]	R1 = 0.0589, wR2 = 0.1769	
R indices (all data)	R1 = 0.0846, wR2 = 0.1919	
Extinction coefficient	n/a	
Largest diff. peak and hole	0.507 and -0.337 e.$Å^{-3}$	
CCDC deposition number	2183452	

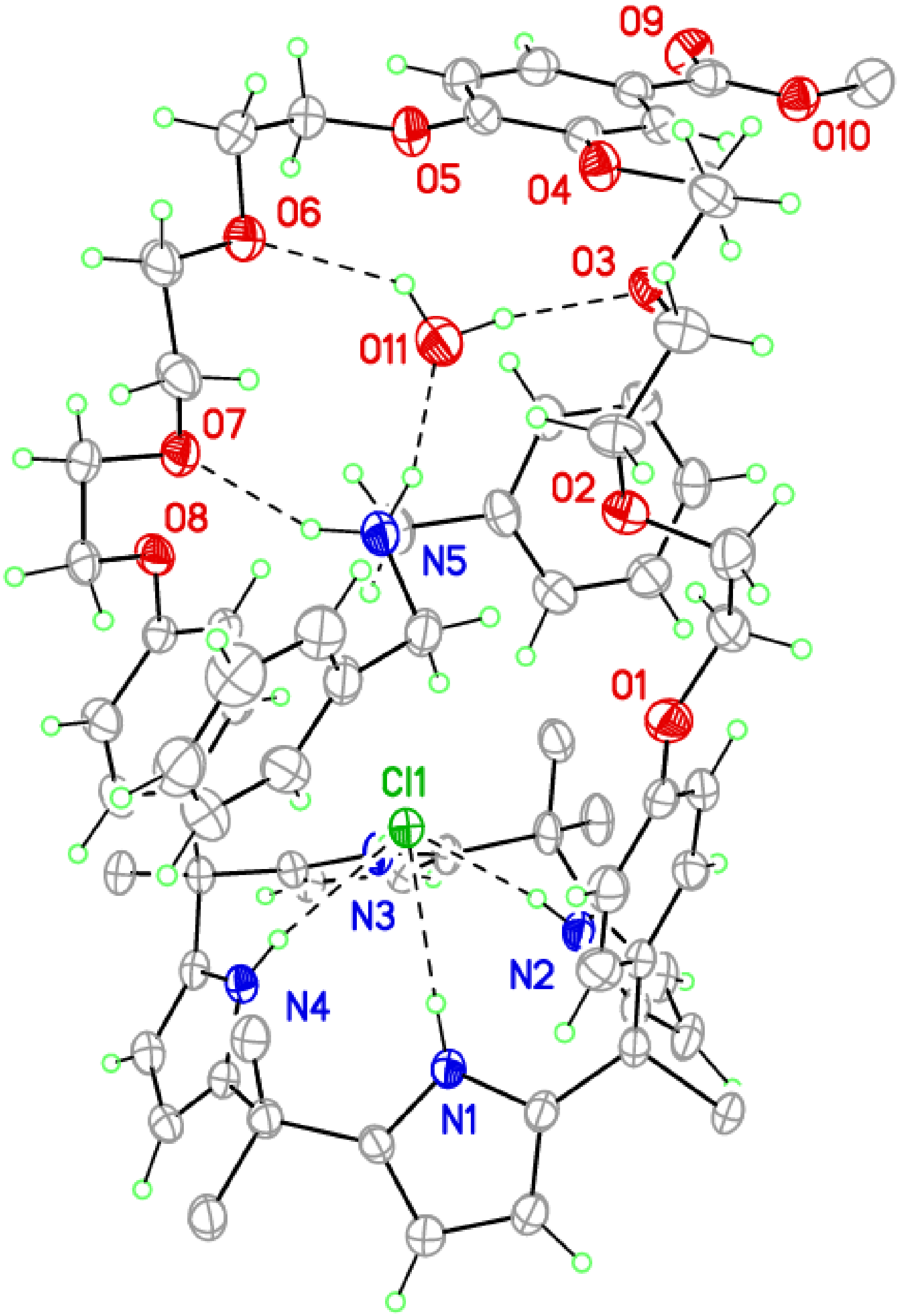

Fig. S30. View of the **G** complex of **H** showing the heteroatom labeling scheme. Displacement ellipsoids are scaled to the 50% probability level. Dashed lines are indicative of inferred H-bonding interactions. The methyl H atoms have been omitted for clarity. The lower occupancy atoms of the disordered methyl ester group are also omitted.

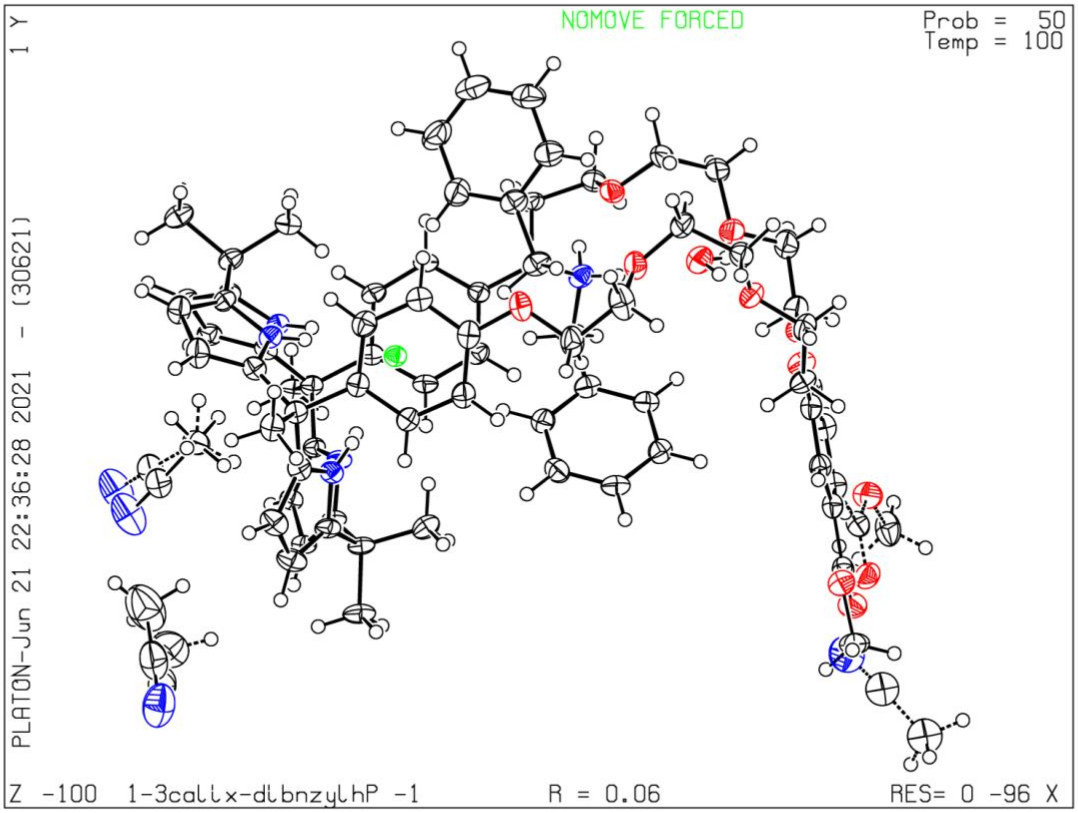

Fig. S31. View of the **G** complex of **H** showing the heteroatom labeling scheme. Displacement ellipsoids are scaled to the 50% probability level. Dashed lines are indicative of an inferred H-bonding interaction. The methyl H atoms have been omitted for clarity. The lower occupancy atoms of the disordered methyl ester group are likewise omitted.

13. Computational methods

The structures were optimized with quantum mechanics / density functional theory (DFT) in the Orca program[S11] (version 4.1.0) at the B3LYP/Def2-SVP level with Grimme's third generation dispersion correction with Becke Johnson damping[S12] (D3BJ). The structures were optimized in three solvent continuum models: SMD, CPCM and COSMO as implemented in Orca. The Cartesian coordinates of all optimized structures are freely available under the CC BY 4.0 license on FigShare from the DOI: https://doi.org/10.6084/m9.figshare.20388369.v1.

14. Computational data

Table S4. Absolute energies (Ha) of ions of interest in acetonitrile continuums.

**Structure**	**Energy / Ha**
Li+_SMD	-7.413819348
Li+_CPCM	-7.457269636
Li+_COSMO	-7.454799
Cl-_SMD	-460.1332007
Cl-_CPCM	-460.147443
Cl-_COSMO	-460.1457749
G+_SMD	-596.9933693
G+_CPCM	-596.9795032
G+_COSMO	-596.9781623

Table S5 Absolute energies (Ha) of the H receptor in acetonitrile continuums.

**Structure**	**Structure in ref S2**	**Energy / Ha**
H_bare_exo_SMD	H2_bare_isomer2_SMD	-3218.413513
H_bare_exo_CPCM	H2_bare_isomer2_CPCM	-3218.398326
H_bare_exo_COSMO	H2_bare_isomer2_COSMO	-3218.395892
H_bare_endo_SMD	H2_bare_isomer3_SMD	-3218.407852
H_bare_endo_CPCM	H2_bare_isomer3_CPCM	-3218.398501
H_bare_endo_COSMO	H2_bare_isomer3_COSMO	-3218.397878

Table S6. Absolute energies (Ha) of the H receptors with bound salts in acetonitrile continuums.

**Structure**	**Structure in ref S2**	**Energy / Ha**
H+LiCl_exo_SMD	H2+LiCl_isomer2_SMD	-3686.153988
H+LiCl_exo_CPCM	H2+LiCl_isomer2_CPCM	-3686.135249
H+LiCl_exo_COSMO	H2+LiCl_isomer2_COSMO	-3686.132941
H+LiCl_endo_SMD	H2+LiCl_isomer3_SMD	-3686.149376
H+LiCl_endo_CPCM	H2+LiCl_isomer3_CPCM	-3686.129537
H+LiCl_endo_COSMO	H2+LiCl_isomer3_COSMO	-3686.128478
H+GCl_exo_SMD	NA	-4275.649889
H+GCl_ exo _CPCM	NA	-4275.629357
H+GCl_ exo _COSMO	NA	-4275.628413
H+GCl_ endo _SMD	NA	-4275.676934
H+GCl_ endo _CPCM	NA	-4275.658389
H+GCl_ endo _COSMO	NA	-4275.656158

Table S7. Binding energies (ΔE_b) of the H receptors with bound salts in acetonitrile continuums.

**Structure**	**Structure in ref S2**	**ΔE_b / kcal mol^{-1}**
H+LiCl_exo_SMD	H2+LiCl_isomer2_SMD	-121.39
H+LiCl_exo_CPCM	H2+LiCl_isomer2_CPCM	-82.96
H+LiCl_exo_COSMO	H2+LiCl_isomer2_COSMO	-85.64
H+LiCl_endo_SMD	H2+LiCl_isomer3_SMD	-122.05
H+LiCl_endo_CPCM	H2+LiCl_isomer3_CPCM	-79.27
H+LiCl_endo_COSMO	H2+LiCl_isomer3_COSMO	-81.59
H+GCl_exo_SMD	NA	-68.90
H+GCl_ exo _CPCM	NA	-56.02
H+GCl_ exo _COSMO	NA	-55.43
H+GCl_ endo _SMD	NA	-89.43
H+GCl_ endo _CPCM	NA	-77.79
H+GCl_ endo _COSMO	NA	-76.39

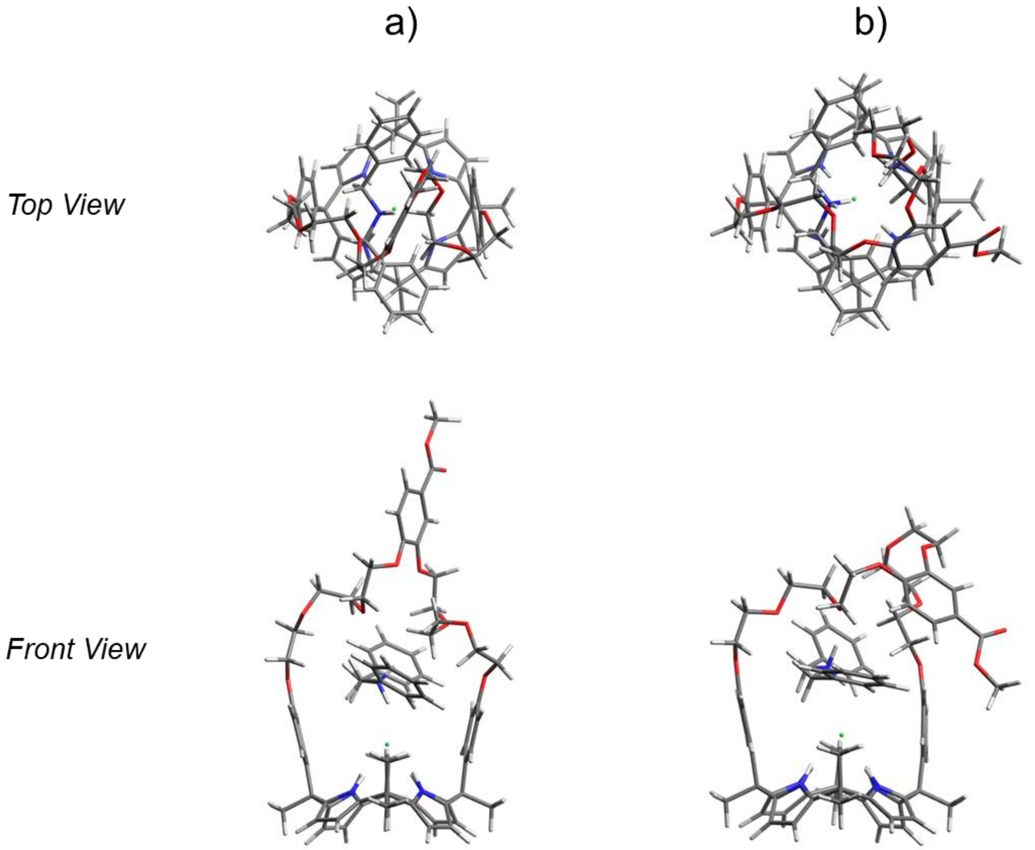

Fig. S32. Calculated geometries (shown in stick representation) of the a) *exo* and b) *endo* conformers with **G** bound; both optimized in the SMD acetonitrile continuum.

15. Supplementary references

S1. R. Søndergaard, S. Strobel, E. Bundgaard, K. Norrman, A. G. Hansen, E. Albert, G. Csaba, P. Lugli, M. Tornow and F. C. Krebs, *J. Mater. Chem.,* 2009, **19**, 3899–3908.

S2. H. Wang, L. O. Jones, I. Hwang, M. J. Allen, D. Tao, V. M. Lynch, B. D. Freeman, N. M. Khashab, G. C. Schatz, Z. A. Page and J. L. Sessler, *J. Am. Chem. Soc.,* 2021, **143**, 20403–20410.

S3. CrysAlisPro. Rigaku Oxford Diffraction (2019). CrysAlicPro Software System, 1.171.41.70a.

S4. G. M. Sheldrick, SHELXT. A Program for Crystal Structure Solution. *Acta Cryst.,* 2015, *A71*, 3-8.

S5. G. M. Sheldrick, SHELXL-2016/6. Program for the Refinement of Crystal Structures. *Acta Cryst.,* 2015, C71, 3-8.

S6. A. L. Spek, PLATON, A Multipurpose Crystallographic Tool. Utrecht University, The Netherlands. *Acta Cryst.,* 2009, D65, 148-155.

S7. O. V. Dolomanov, L. J. Bourhis, R. J. Gildea, J. A. K. Howard and H. Puschmann, OLEX2. A Complete Structure Solution, Refinement and Analysis Program. *J. Appl. Cryst.,* 2009, **42**, 339-341.

S8. $R_W(F^2) = \{\Sigma w(|F_O|^2 - |F_C|^2)^2 / \Sigma w(|F_O|)^4\}^{1/2}$ where w is the weight given each reflection. $R(F) = \Sigma(|F_O| - |F_C|)/\Sigma|F_O|\}$ for reflections with $F_O > 4(\sigma(F_O))$. $S = [\Sigma w(|F_O|^2 - |F_C|^2)^2 /(n - p)]^{1/2}$, where n is the number of reflections and p is the number of refined parameters.

S9. International Tables for X-ray Crystallography (1992). Vol. C, Tables 4.2.6.8 and 6.1.1.4, A. J. C. Wilson, editor, Boston: Kluwer Academic Press.

S10. G. M. Sheldrick, SHELXTL/PC (Version 5.03). 1994. Siemens Analytical X-ray Instruments, Inc., Madison, Wisconsin, USA.

S11. F. Neese, The ORCA Program System. *Wiley Interdiscip. Rev.: Comput. Mol. Sci.,* 2012, **2**, 73-78.

S12. S. Grimme, S. Ehrlich and L. Goerigk, Effect of the damping function in dispersion corrected density functional theory. *J. Comput. Chem.,* 2011, **32**, 1456-1465.
